# mTORC1-signaling switches megalin function from endocytosis to cell cycle progression

**DOI:** 10.1007/s00018-026-06247-5

**Published:** 2026-05-23

**Authors:** Eileen Dahlke, Madlen Kunke, Hannah Knöfler, Yaman Anan, Yuanhao Shen, Danyang Zhao, Christine von Toerne, Franziska Theilig

**Affiliations:** 1 Institute of Anatomy, Christian Albrechts-University Kiel, Otto-Hahn-Platz 8, Kiel, 24118 Germany; 2 Metabolomics and Proteomics Core, Helmholtz Center Munich, German Research Center for Environmental Health, D-80939 Munich, Germany

**Keywords:** LRP2, Metaphase, Proteolytical cleavage, Clathrin, Kidney, Proximal tubule

## Abstract

Mechanistic target of rapamycin (mTOR) signaling pathway controls eukaryotic growth by regulating metabolism, translation, autophagy and cell cycle. Genetic deletion of renal mTORC1 led to a Fanconi-like syndrome with reduction of the renal cortex, tubular epithelial transport and perturbation of the endocytic machinery. Although the main scavenger receptor megalin remained unaltered, a new phosphorylation site at S4577 was found. The identification of the role of this mTORC1-induced phosphorylation on megalin was subject of our analysis. mTORC1-induced megalin phosphorylation reduced endocytosis rate with only minor distribution changes. It augmented the affinity of megalin to the adaptor protein ARH with consecutively increased proteolytic processing of megalin C-terminal domain and favored cell proliferation. During cell mitosis megalin is localized with ARH at the spindle pole. Compared to wildtype cells, megalin-deficient and ARH-deficient cells showed significantly less cell proliferation, and during cytokinesis significantly less ARH or megalin signals, respectively at the pole of intercellular bridges. mTORC1-induced megalin S4577 phopshorylation varies throughout the cell cycle with highest abundance in metaphase and telophase/cytokinesis. Both, lysosomal and non-lysosomal (external) nutrient supply influence cell proliferation to different extent. In conclusion, absence of the mTORC1-induced megalin S4577 phosphorylation favors cell growth and clathrin-mediated endocytosis whereas mTORC1-induced megalin phosphorylation at S4577 favors cell proliferation and increases the affinity to the adaptor protein ARH for proper cell division. Especially, during cytokinesis megalin and ARH ensure trafficking of membrane vesicles towards the pole of intercellular bridges most probably for the terminal abscission process.

**Supplementary Information:**

The online version contains supplementary material available at 10.1007/s00018-026-06247-5.

## Introduction

The mechanistic target of rapamycin (mTOR) signaling pathway controls eukaryotic growth by integrating environmental with intracellular cues [1]. MTOR complex 1 (mTORC1) is a central signaling hub between anabolic and catabolic processes with established roles in regulating metabolism, translation, autophagy and cell cycle progression. Hyperactivity of mTOR1 is associated with human diseases such as diabetes mellitus or cancer. Pharmacological inhibition of the mTORC1 pathway is an approved therapy for immunosuppression after organ transplantation by reducing viral infections and neoplastic complications [2, 3]. In the kidney, mTORC1 is one of the master regulators of renal epithelial transport and endocytosis [4]. Inhibition or genetic deletion of renal mTORC1 leads to Fanconi-like syndrome with reduction of the renal cortex due to shrinkage of the proximal convoluted tubule, perturbation of the endocytic machinery and tubular transport. As underlying cause reduced expression or loss of phosphorylation at critical residues of specific transport proteins were identified. Although the expression of the main endocytic scavenger receptor megalin (also called low-density lipoprotein-related protein 2) involved in clathrin-mediated endocytosis remained unaltered, reduced endocytosis together with loss of megalin phosphorylation at the megalin C-terminus S4577 was observed. Megalin is expressed predominantly in renal proximal tubule epithelial cells, as well as in the brain, lungs, eyes, inner ear, thyroid gland, testis, and placenta [5]. Mutations of the megalin gene (*LRP2*) cause Donnai-Barrow syndrome (facio-oculo-acoustico-renal syndrome) in humans. Megalin is composed of a large extracellular domain for ligand binding, a transmembrane domain and an intracellular cytoplasmic domain for its regulation. The latter therefore possesses two NPxY binding motifs for the adaptor proteins disabled-2 (Dab2) and autosomal recessive hypercholesteremia (ARH) coupling the receptor to the clathrin machinery [6]. ARH binding was further shown to guide megalin to the endosomal recycling complex thereby inhibiting the fast-recycling pathway from the early endosomes directly to the plasma membrane. Megalin further exhibits at the cytoplasmic domain a PPPSP motif which is constitutively phosphorylated by GSK3β accounting most significantly for basal phosphorylation of the receptor [7] independent to ligand binding. Phosphorylation of the PPPSP motif negatively regulates megalin recycling without altering basal endocytosis rate [7]. The aim of this study is to identify to what cellular purpose mTORC1 signaling involves megalin and phosphorylates megalin at its C-terminal part S4577. Therefore, megalin mutants S4577D and S4577A, mimicking either phosphorylation or absent phosphorylation at this site respectively, were generated and analyzed for mTORC1-induced cell functions such as clathrin-mediated endocytosis and cell cycle progression.

## Materials and methods

### Animals, fixation and tissue processing for immunohistochemistry

Transgenic mice with targeted renal tubular deletion of von-Hippel-Lindau (VHL^ΔTub^) had been generated as described in Theilig et al. [8] by cross-breeding Pax8–reverse tetracycline-dependent transactivator mice, expressing the reverse tetracycline-dependent transactivator under control of the Pax8 promoter with LC-1 mice, expressing Cre recombinase under the control of the bidirectional P_tet_ promoter with VHL^flox/flox^ mice for targeted disruption of VHL.

Mice were anesthetized and kidneys were perfused retrogradely using 4% PFA in PBS. Perfusion-fixed specimens were postprocessed for cryo- or paraffin-embedding for further histochemical light microscopy analysis.

All animal experiments were conducted according to the National Institutes of Health guide for the care and use of laboratory animals as well as the Swiss law for the welfare of animals, and they were approved by the Cantonal Veterinary Office (Canton of Fribourg, Switzerland) (FR25959, 2014_55_FR). All protocols were reviewed by the University’s Animal Welfare and Ethics Review Board before experimentation.

## Cell culture, transient transfection and generation of megalin-deficient cells and megalin mini-receptor mutants

HEK293 (Human Embryonic Kidney cells), MDCK-II (Madin-Darby canine kidney cells) sharing some characteristics of proximal tubule cells [9], OKC (Opossum kidney cells) [10, 11] and HK2 (immortalized human kidney cells) [11, 12] proximal tubule cells, and BN16 (Brown Norway rat yolk sac epithelial cells) with endogenous high expression of megalin [13] were used and incubated at 37 °C in 5% CO_2_/95% air atmosphere. HEK293 were cultures in Dulbecco’s modified Eagles medium high glucose (DMEM, P04-03590, PAN Biotech, Aidenbach, Germany), 10% fetal calf serum (PAN Biotech), 100 U/ml penicillin (PAN Biotech), 100 µg/ml streptomycin (PAN Biotech). MDCKII were cultured in Alpha MEM (LONZA, Köln, Germany), 10% fetal calf serum, 2 mM L-glutamine (Sigma Aldrich), 100 U/ml penicillin, 100 µg/ml streptomycin. OKC were cultured in a composition of 1:1 mixture of Dulbecco’s modified Eagles medium (DMEM; PAN Biotech) and Ham’s F-12 (P04-15500, PAN Biotech) supplemented with 10% fetal bovine serum, 2 mM glutamine, 100 U/ml penicillin, 100 µg/ml streptomycin, 3.0 g/l Na_2_CO_3_ and 5 mM HEPES. HK2 were cultured in a composition of 1:1 mixture of Dulbecco’s modified Eagles medium (DMEM, 25 mM glucose; Sigma Aldrich) and Ham’s F-12 (P04-15500, PAN Biotech) supplemented with 10% fetal bovine serum, 2 mM glutamine, 100 U/ml penicillin, 100 µg/ml streptomycin. BN16 cells were cultured in DMEM supplemented with 10% fetal calf serum (PAN Biotech) and 100 U/ml penicillin, 100 µg/ml streptomycin.

Generation of megalin- and ARH-deficient BN16 cells. Megalin and ARH knockout was generated in rat BN16 cells by the CRISPR/Cas9 genome editing technology using the Gene knockout kit v2 (Synthego). Three different sgRNAs targeting exon 2 of the rat megalin gene or exon 2 of rat ARH gene were designed by Synthego and diluted at a concentration of 60 pmol/µl. BN16 cells were transfected with sgRNAs in presence of CAS9 nuclease by electroporation using the Neon transfection system (Thermo Fisher Scientific) following the recommendation by the manufacturer. After 72 h, cells were plated at glass cover slips for immunohistochemistry. Wildtype and megalin- or ARH-deficient cells were analyzed in a side-by-side dependent manner using the established megalin antibody [14–17] or a commercial ARH antibody to define cell type.

Transient transfection. For transient transfection, cells were plated on glass coverslips or culture dishes covered with poly-D-lysine and transfected the day after using either calcium-phosphate buffer, X-fect™ (Takara Bio Group, Kusatsu, Japan) or JetOptimus^®^ (Polyplus, Illkirch-Graffenstaden, France). If not stated otherwise, 72 h after transfection, cells were used for endocytosis assay or lysed using RIPA buffer supplemented with protease inhibitor cocktail (Complete EDTA-free Protease Inhibitor, 11873580001, Roche Diagnostics, Mannheim, Germany) and phosphatase inhibitors (10 mM sodium pyrophosphate, 100 mM sodium fluoride, 1 mM sodium orthovanadate).

**Fig. 1:**
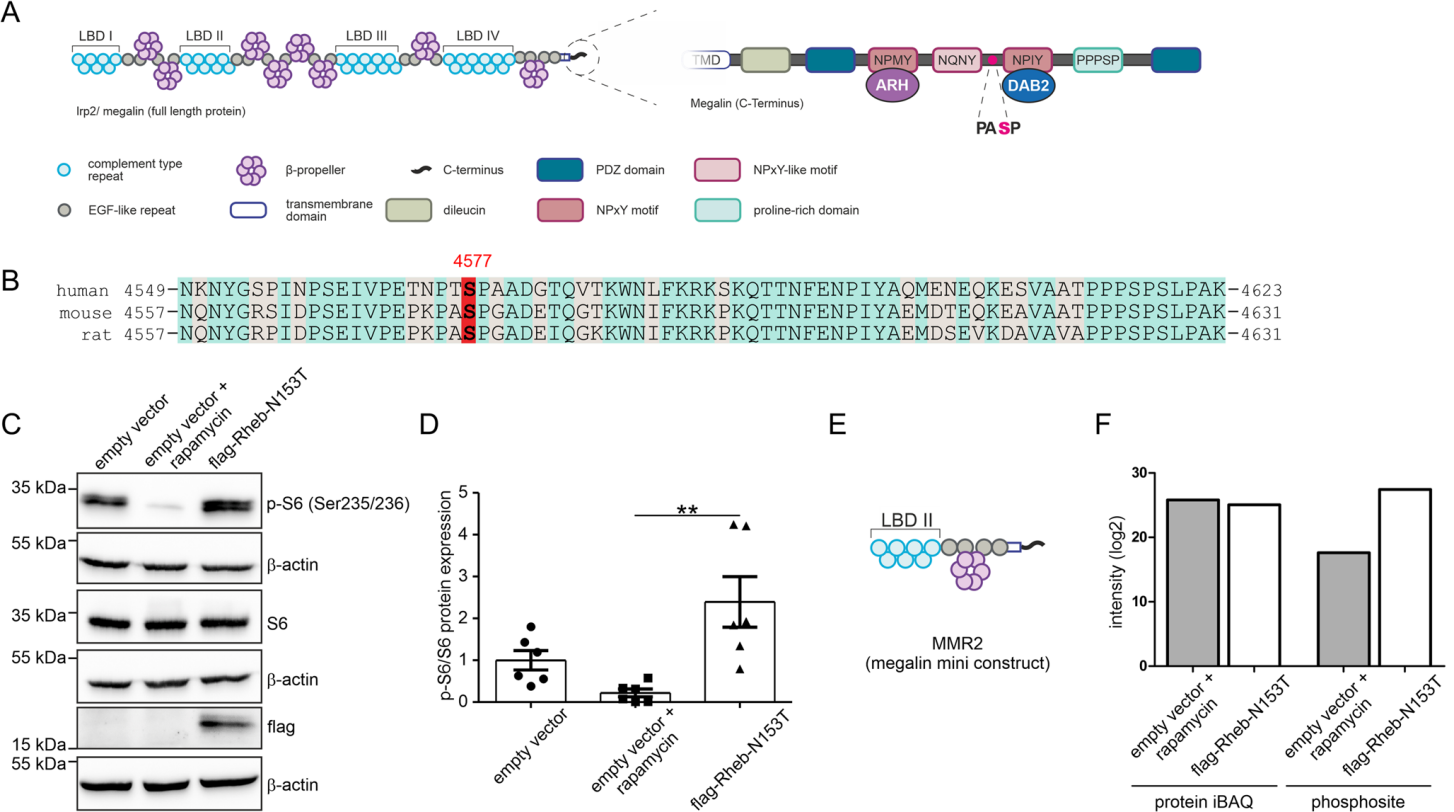
mTORC1 phosphorylates megalin at S4577 in its C-terminus. (**A**) Scheme of megalin showing its structural elements. Higher magnification of megalin C-terminus is shown on the right depicting the new phosphorylation site of serine 4577 (S4577, S marked in red color). (**B**) Comparison of amino acid sequence of megalin C-termini from human, mouse and rat showing the conservation of the phosphorylation site S4577 across species. Identical amino acids were marked in light blue, differences in beige and S4577 is highlighted in red. (**C** and **D**) Western blot and densitometric evaluation of p-S6 normalized to protein S6 and β-actin upon mTORC1 activation by Rheb-N153T or by mTORC1 inhibition with 100 nM rapamycin for 3 h in HEK293 cells. Values are mean ± SEM. Kruskal-Wallis test followed by Dunns post test, ** *P* < 0.01. (**E**) Scheme of megalin mini-receptor construct 2 (MMR2) used in experiments, including ligand-binding domain 2 (LBD II), transmembrane domain and C-terminal part. (**F**) Total protein content (left, protein iBAQ) and quantitative analysis of phosphorylation intensity on megalin S4577 (right) of HEK293 cells transfected with MMR2 under mTORC1 activation (flag-Rheb-N153T transfection) or mTORC1 inhibition (empty vector + rapamycin treatment)

Megalin mini-receptor mutants. Megalin mini-receptors are a broadly accepted tool to analyze endocytosis in transfected cells. Our vectors included the coding sequence of the ligand binding domain 2, the transmembrane domain and the C-terminal part of the receptor (for details, see Fig. 1). To study to impact of the phosphosite S4577, vectors carrying mini-megalin receptor coding sequence were mutated to generate an amino acid exchange from S ◊ A (mimics no phosphorylation) and S ◊ D (mimics phosphorylation).

##  mTORC1 activation/inhibition and mass spectrometry ofmegalin mini-receptor

HEK293 cells were transiently transfected megalin mini-receptor and either additionally with RhebN153T (Addgene [18]), for mTORC1 activation or incubated with 100 nM rapamycin, a selective mTORC1 inhibitor, 3 h before the end of experiment. Megalin mini-receptor were co-immunoprecipitated and separated on a SDS-PAGE gel. After Coomassie staining protein bands were cut out and analyzed per mass spectrometry at the CECAD multi user facility for mass spectrometry University of Cologne, Germany. Total protein levels were similar and were shown as IBAQ (intensity-based absolute quantification) values.

## Pulse-chase and steady state endocytosis assay

To enhance cell differentiation/polarization to a more physiological phenotype MDCK-II cells were seeded on petri dishes in growth medium and cultured under standard conditions. After 24 h, MDCK-II cells were transiently transfected and cultured on an orbital shaker at 1 Hz shear conditions for another 72 h prior pulse-chase experiment. Differentiation of cells was controlled by the appropriate expression of the tight junction marker ZO-1. HK2 cells were seeded in standard growth medium and transiently transfected the day after. 48 h after transfection, cells were incubated in differentiation medium (DMEM low glucose (5.5 mM glucose, Sigma Aldrich), 2% FCS, 2 mM glutamine, 100 U/ml penicillin, 100 µg/ml streptomycin).

Pulse-chase endocytosis assay. Polarized MDCK-II cells or HK2 cells were starved for 6 h and placed on ice, washed with ice-cold PBS and incubated with AlexaFluor647-coupled albumin (0.2 mg/ml) for 30 min on ice (pulse). After extensive washing with ice-cold PBS cells were placed at 37 °C for 15 min (chase). Afterwards cells were placed on ice, washed several times and fixed with 4% PFA/PBS for further immunocytochemical analysis.

Steady state endocytosis assay. Polarized MDCK-II cells or BN16 cells were starved for 6 h, washed with cold PBS and incubated with albumin (0.2 mg/ml) for 5–15 min at 37 °C, respectively. Cells were placed on ice, washed with ice-cold PBS, fixed with 4% PFA/PBS for further immunocytochemical analysis.

## Cell cycle synchronization

To analyze the BN16 cells in a specific cell cycle phase, cells were synchronized. For G1 phase, completely confluent and differentiated BN16 were used. To synchronize cells in the late telophase/beginning cytokinesis, we used a double thymidine block in addition with nocodazole treatment. In detail, cells were plated in a density of 10%. 14 h later, cells were treated with 2 mM thymidine (HY-N1150, Hölzel Diagnostika Handels GmbH, Cologne, Germany) in normal growth media for 18 h, before cells were washed twice with PBS and incubated in normal growth media for 8 h. The procedure was repeated once. 9 h after the second release, cells were treated with 20 ng/ml nocodazole (HY-13520, Hölzel) in DMEM medium (without serum) for 5 h. Cells were washed twice with PBS and released in normal growth media for 65 min. To synchronize the cells in the metaphase, a double thymidine block and nocodazole treatment was used (as described above) with an additional treatment of MG-132. In detail, direct after the nocodazole treatment, cells were washed twice in PBS and treated with 25 µM MG-132 (S2619, Selleckchem, Cologne, Germany) in DMEM medium (without serum) for 70 min. Cells were washed in PBS and released in normal growth medium for 30 min.

### Immunocyto- and immunohistochemistry

Cells or 5 μm renal cryo-sections were permeabilized using 0.5% tritonX-100/PBS for 30 min, blocked with 5% skim milk/PBS for 1 h and incubated with primary antibody overnight at 4 °C. After washing, samples were incubated with suitable secondary antibodies for 2 h at room temperature. Nuclei were stained using 4’,6-diamidino-2-phenylindole (DAPI, Cat # D1306, Thermo Fisher) and mounted with Abberior Mount Liquid Antifade (MM-2009-2 × 15ML, Abberior GmbH, Göttingen, Germany). Images were either acquired using Facility Line (Abberior Instruments, Göttingen, Germany) with Olympus IX83 microscope (Hamburg, Germany), 100x objective (NA 1.4 oil) or 40x objective (NA 1.3 oil) and Imspector software (Abberior Instruments, Göttingen, Germany) or BZx800 microscope (Keyence, Frankfurt am Main, Germany). Confocal and STED images were de-convoluted using Huygens Professional Software (version 20.10, Scientific Volume Imaging B.V., Hilversum, The Netherlands). CMLE algorithm using a quality change threshold of 0.1 and a maximum of 40 cycles was performed. For co-localization analysis the colocalization analyzer of Huygens software were used and mean values of the Pearson correlation coefficient (PCC) were evaluated statistically. Fluorescence signal intensity were evaluated using ImageJ software.

For cell surface analysis, fixed cells were stained with 10 µg/ml WGA-AlexaFluor488 (Wheat Germ Agglutinin, W11261, ThermoFisher Scientific) for 30 min at room temperature and washed five times before cells were permeabilized using 0.5% tritonX-100/PBS and double stained with megalin as described above.

## Antibodies

The following antibodies were used: guinea pig anti-megalin [14], rabbit anti-clathrin (CBL188, Millipore, Darmstadt, Germany), rabbit anti-clathrin (ab21679, Abcam, Cambridge, UK), mouse anti-EEA1 (#610457, BD Biosciences, Heidelberg, Germany), rabbit anti-Rab11 (71–5300, Thermo Fisher Scientific/Invitrogen, Munich, Germany), rat anti-ZO-1 (Millipore, R40.76; MABT11), mouse anti-β-actin (sc-47778, Santa Cruz), mouse anti-GST (G1160, Sigma-Aldrich, Steinheim, Germany), mouse anti-myc (clone 9E10, M4439, Sigma-Aldrich), rabbit anti-myc (10828-1-AP, Proteintech, Planegg-Martinsried, Germany), rabbit anti-Rab35 (11329-2-AP, Proteintech), rabbit anti-Ki67 (MA5-14520, Thermo Fisher Scientific/Invitrogen), rat anti-Ki67 (#14–5698-82, Thermo Fisher Scientific), rabbit anti-ARH (# bs-6337R, Bioss Antibodies, USA), mouse anti-alpha-Tubulin (66031-1-Ig, Proteintech), rabbit anti-S6 (#2217, Cell Signaling Technology, Danvers, Massachusetts, USA), rabbit anti-phospho-S6 (Ser235/236) (#2211, Cell Signaling Technology), rabbit anti-phospho-TFEB (Ser122) (#87932, Cell Signaling Technology), rabbit anti-TFEB (A303-673 A, Biomol, Hamburg, Germany), rabbit anti-Histone 3 (17168-1-AP, Proteintech), rabbit anti-His tag (GSC-A00174, Biomol).

Secondary antibodies used were coupled with StarRed, Star580 or AlexaFluor488 and either purchased from Abberior GmbH (anti-rabbit Star580 (ST580-1002); anti-mouse Star580 (ST580-1001), anti-guinea pig StarRed (STRED-1006)) or donkey-derived secondary antibodies (Dianova) were coupled with Star580 or StarRed as described [13].

## Inhibition of proteasomal digestion

To study regulated intramembrane proteolysis, HEK293 cells were transiently transfected with mini-megalin receptor mutants and 48 h later treated with MG-132 (a potent, reversible, and cell-permeable proteasome inhibitor, 10 µM, S2619, Selleckchem, Cologne, Germany) overnight followed by cell lysis with RIPA buffer and protease inhibitor cocktail (Complete EDTA-free Protease Inhibitor, 11873580001, Roche Diagnostics, Mannheim, Germany) or using NucleoSpin RNA/Protein isolation kit (740933.50, Macherey-Nagel, Düren, Germany) according to manufacturer’s instructions.

### Bacterial expression vectors, protein production and pulldown assay

E. coli were transfected with vectors coding for GST protein (pGEX4T-3; GE Healthcare, München, Germany), GST-tagged Dab2 (pGEX4T1-GST-DAB2_1−205_ coding for mouse Dab2 amino acids 1–205 with N-terminal GST tag; [19]) and GST-tagged ARH (pGEX-2T-hLDLRAP1 coding for human ARH with N-terminal GST tag; Vectorbuilder), respectively. Protein expression was induced using 0.1 mM IPTG (isoprolyl-β-D-thiogalactopyranosid, Roth, Karlsruhe, Germany).

For expression of megalin C-terminal S4577 wildtype and mutants S◊A and S◊D proteins, E. coli were transfected with pET-6xHis/Megalin C-term_600bp WT, pET-6xHis/Megalin C-term_600bp SA and pET-6xHis/Megalin C-term_600bp SD, respectively (LPSLSSLAKPSENGNGVTFRSGADVNMDIGVSPFGPETIIDRSMAMNEHFVMEVGKQPVIFENPMYAAKDNTSKVALAVQGPSTGAQVTVPENVENQNYGRPIDPSEIVPEPKPASPGADEIQGKKWNIFKRKPKQTTNFENPIYAEMDSEVKDAVAVAPPPSPSLPAKASKRNLTPGYTATEDTFKDTANLVKEDSDV*). Protein expression was induced using 1 mM IPTG.

Proteins were isolated in PBS containing protease inhibitor (Complete EDTA-free protease inhibitor, Roche Diagnostics). After cell lysis by sonification, glutathione agarose beads were used to isolate GST-tagged proteins from the lysate. For the purification of His-tagged proteins Ni-NTA agarose beads were used and isolated proteins were dialyzed against PBS.

1 µg of purified GST protein or GST-tagged ARH and DAB2_1−205_ proteins were diluted in 2 ml PBS incubated with 25 µl Glutathione beads for 1 h on rotator at 4 °C. After washing in RIPA buffer, 10 µg of purified megalin C-terminal mutants added and incubated for another hour at 4 °C. After washing in RIPA buffer, proteins were eluted from beads using Laemmli buffer, denaturated at 95 °C for 10 min and used for SDS-PAGE.

### Differential mTORC1 activation

To differentially evaluate the effect of lysosomal and non-lysosomal mTORC1 signaling on cell proliferation, we used OKC and treated them with different media conditions for 24 h before harvesting and protein isolation or fixation and immunocytochemical staining. Analogous to Fernandes et al. [20], we used [1] growth media containing amino acids (DMEM/Ham’s F12) [2], growth media containing amino acids and supplemented with pepstatin A (50 µM, #2936, Roth) and E-64 (25 µM, HY-15282, MedChem Express, Sollentuna, Sweden) to block lysosomal mTORC1 [3], growth media lacking amino acids (D9807-11, US Biological, Salem, Massachusetts, USA) to block non-lysosomal mTORC1, and [4] growth media lacking amino acids supplemented with pepstatin A and E-64 to block all mTORC1. After 24 h, cytoplasmic and soluble nuclear proteins were isolated using Nuclear Extraction Kit (ab219177, Abcam, Cambridge, UK) according to manufacturer’s instructions.

### SDS-PAGE and immunoblotting

Isolated proteins were separated using SDS gel electrophoresis on polyacrylamide gels and transferred electrophoretically onto nitrocellulose membranes. Protein loading were verified by staining the membrane with 0.1% Ponceau red. Membranes were blocked with 5% skim milk/TBS and incubated with primary antibodies overnight. After incubation with HRP-conjugated secondary antibodies (Dianova, Hamburg, Germany), immunoreactive bands were detected by chemiluminescence using Immobilon Western HRP substrate (Millipore, Darmstadt, Germany) and chemiluminescence imaging system Fusion SL (Peqlab, Erlangen, Germany). Signal density were analyzed using ImageJ software.

### Immunoprecipitation and sample preparation for mass spectrometry of endogenous megalin

For immunoprecipitation, membrane proteins were isolated from BN16 cells conditioned in G1-phase, metaphase and late telophase in triplicates, and enriched using 0.25 mol/L sucrose buffer supplemented with protein inhibitor cocktail (cOmplete, EDTA-free, Roche Diagnostics) and phosphatase inhibitors (10 mM sodium phosphate, 100 mM sodium fluoride, 1 mM sodium orthovanadate). As negative control, cells in G1-phase were used. Magnetic beads (Pierce protein A/G magnetic beads, Thermo Fisher) were incubated with 5 µl guinea pig anti-megalin [14], or 5 µl anti-His tag for the negative control and 450 µg of isolated membrane proteins for 4 h at 4 °C, washed and bound protein was eluted with Laemmli buffer at for 5 min at 95 °C. Samples were separated on an 8% SDS gel and gel was stained with Coomassie brilliant blue for 30 min and unstained using 20% ethanol/10% acidic acid overnight. The megalin bands at 600 kDa as well as the negative control at the same height were cut out. The cut gel pieces were enzymatically digested using trypsin overnight. Acetonitrile eluted peptides were acidified and speedvac-dryed until mass spectrometric analysis.

### Mass spectrometric measurement of endogenous megalin

Peptides were dissolved in 0.01% n-Dodecyl-D-Maltoside in 1% FA and loaded on Evotips (one Evotip for each injection) and run on an Evosep One LC (Evosep, Odense, Denmark). The 40 samples per day whisper method employing a 27 min gradient with solvents A (0.1% FA, H2O) and B (0.1% FA, MeCN) was chosen and a 15 cm column (AuroraElite C18, 1.7 μm beads, 150 μm ID) used for separation of peptides. The samples were measured on a TIMS quadrupole TOF mass spectrometer (Bruker timsTOF Ultra2) in DIA-PASEF mode with TIMS on. The default diaPASEF method from Bruker was used (‘diaPASEF – low sample amount’) with a scan range of m/z 100 to 1,700 and mobility range of 0.64–1.45 1/K0. A duty cycle of 100% was achieved by setting the ramp time and accumulation time to 100 ms each. Cycle time was 0.96 s.

### Data analysis

Spectra were analyzed using DIA-NN (version 2.3 academia [21]), using a Swissprot rat database (release 2020_02 consisting of 8062 sequences). Standard settings were used with the following exceptions: phospho (STY) was allowed as variable modification and MS1 and MS2 accuracy were manually set to 10. Phospho site localization filtering was 0.9. Relative abundances from phospho peptides were used for quantification and comparison across conditions. The mass spectrometry proteomics data have been deposited to the ProteomeXchange Consortium via the PRIDE [22] partner repository with the dataset identifier PXD071589.

### Statistics

Statistical comparisons were performed with the GraphPad Prism Software Package 5 (GraphPad Software, La Jolla, CA, USA) using the Kruskal-Wallis test following Dunns post test or Mann-Whitney U test. P values of < 0.05 were judged statistically significant. Asterisks are used in the figures to explicitly demonstrate the statistical significance (* *P* < 0.05; ** *P* < 0.01).

## Results

### mTORC1 phosphorylates megalin at S4577 of its cytoplasmic domain

In our previous phosphoproteom analysis of renal cortices obtained from *Raptor*-deficient mice with consecutive mTORC1 (mammalian target of rapamycin complex 1) ablation [4], we identified a new phosphorylation site of megalin within the C-terminal (intracellular) part of the protein at serine 4577. It is situated between the two NPxY motifs (Fig. 1A). This phosphorylation site is conserved across species as shown for human, mouse and rat (Fig. 1B). It may therefore have an important impact on the expression level, distribution or function of megalin. To confirm previous results on mTORC1-induced phosphorylation of megalin, HEK293 cells were used and mTORC1 was activated or inhibited by transient transfection of Rheb-N153T [18], a hyperactive variant of the mTORC1 activator Rheb, or treated with 100 nM rapamycin, respectively (Fig. 1C). To normalize transfection condition, control and rapamycin-treated cells received empty vector transient transfection. Downstream phosphorylation of protein S6 was used to confirm successful mTORC1 activation/inhibition (Fig. 1C, D). mTORC1 activation in Rheb-N153 transfected cells demonstrated significantly enhanced phosphorylation of protein S6, which was nearly absent in rapamycin-treated cells. To proof, that mTORC1 activation leads to S4577 phosphorylation, cells were co-transfected in addition with megalin mini-receptor constructs (MMR2, Fig. 1E), followed by immunoprecipitation and phosphoproteomic analysis. Phosphorylation on megalin S4577 was 900-fold increased when mTORC1 was activated in comparison to mTORC1 inhibition (Fig. 1F). Protein concentration determined by iBAQ (intensity-based absolute quantification) remained unaltered. 

### mTORC1-induced phosphorylation on megalin S4577 reduces ligand endocytosis and mildly changes the intracellular distribution of megalin

**Fig. 2:**
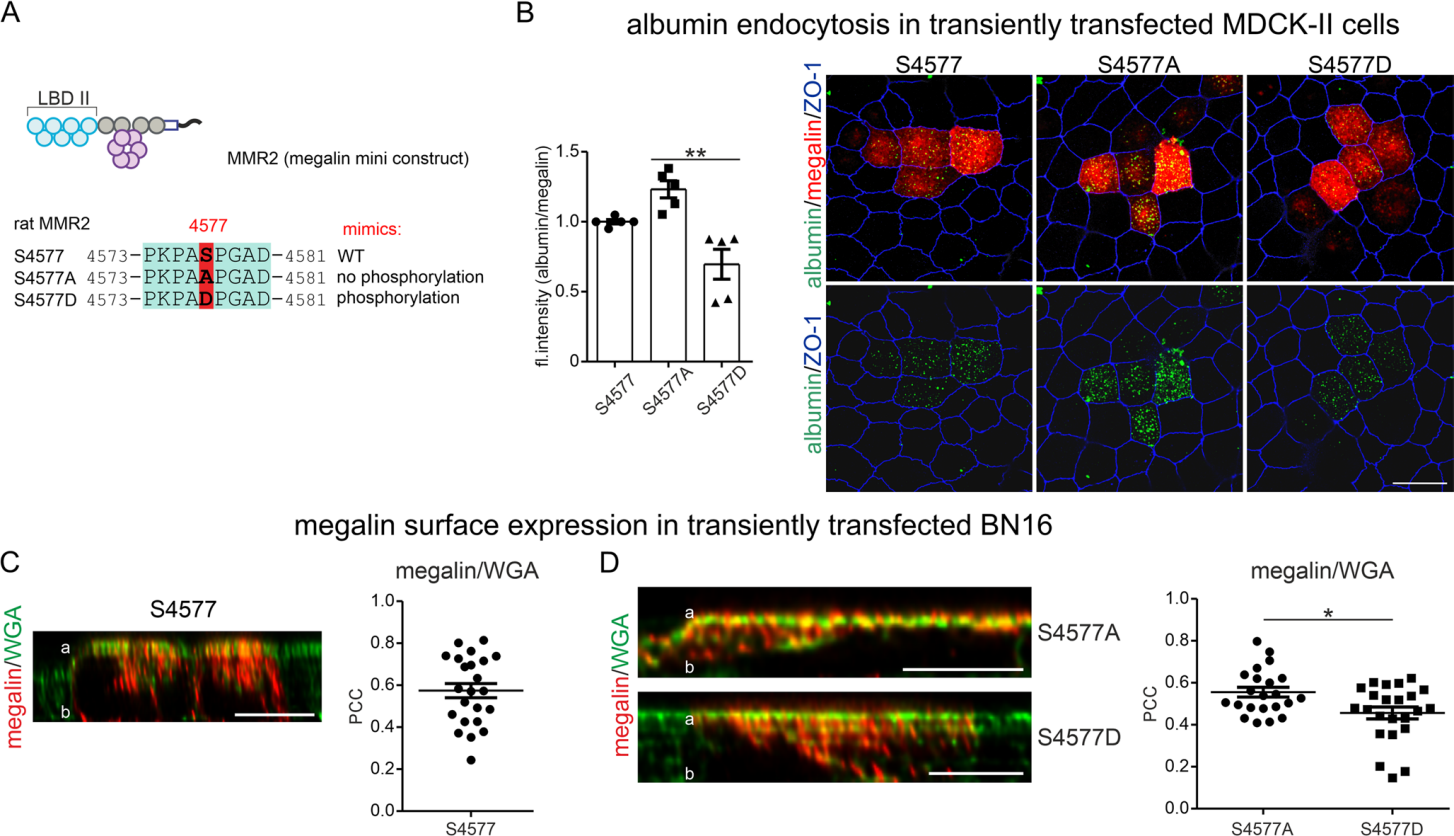
mTORC1-induced S4577 phosphorylation reduces albumin uptake and alters intracellular distribution of megalin. (**A**) Scheme of megalin mini-receptor construct 2 (MMR2) and mutated versions. In S4577A, serine is mutated to alanine mimicking no phosphorylation in C-terminus; in S4577D, serine is mutated to aspartate mimicking permanent phosphorylation. (**B**) Quantification and representative graphs depicting albumin uptake of megalin mutants in transiently transfected MDCK-II cells. Representative images of albumin uptake are shown in green. Cells were co-stained with anti-megalin (red) for identification of mutant transfected cells and with anti-ZO-1 to mark tight junctions/cell borders for verification of cellular differentiation. Scale bar = 20 μm. Values are mean ± SEM. Kruskal-Wallis test followed by Dunns post test, ** *P* < 0.01. (**C**) Confocal imaging of a z-axis of MMR2 S4577 transfected BN16 cells stained with anti-megalin in red and the cell surface with wheat germ agglutinin (WGA)-AlexaFluor488 in green. Quantitative analysis of Pearson correlation coefficient (PCC) of megalin MMR2 S4577 with WGA showing a pronounced colocalization and therefore surface expression. >20 cells of *n* = 3 independent experiments were evaluated. Scale bar = 5 μm. a, apical site; b, basal site. (**D**) Confocal imaging of a z-axis of MMR2 S4577A and S4577D transfected BN16 cells stained with anti-megalin in red and the cell surface with WGA- AlexaFluor488 in green. Quantitative analysis of PCC of megalin MMR2 S4577A or MMR2 S4577D with WGA showing a significant higher colocalization of MMR2 S4577A with WGA compared to MMR2 S4577D with WGA. >20 cells of *n* = 3 independent experiments were evaluated. Scale bar = 5 μm. a, apical site; b, basal site. Mann-Whitney-U test, *P < 0.05

MMR2 plasmid was mutated at S4577 to generate constructs mimicking the non-phosphorylated state (serine 4577 mutated to alanine, S4577A; Fig. 2A) and mimicking permanent phosphorylation (serine 4577 mutated to aspartate, S4577D). These constructs were used to analyze the impact of this phosphorylation site on megalin-dependent endocytosis and its cellular distribution. To assess the endocytosis rate, the proximal tubule like MDCK-II cells without endogenous megalin expression, were transfected with either MMR2 S4577 or S4577A or S4577D, respectively. Pulse-chase albumin uptake using AlexaFluor555-coupled albumin performing 30 min pulse at 4 °C and chase for 15 min at 37 °C revealed in cells transfected with S4577D a significantly reduced albumin uptake in comparison to S4577A (Fig. 2B). A Pearson correlation analysis showed a significant correlation between megalin expression levels in transfected cells and the amount of endocytosed albumin (Figure S1). These results observed in MDCK-II cells were confirmed by using a human proximal tubule cell line HK2, showing reduced albumin uptake in S4577D compared to S4577A transfected cells (Figure **S1B**, **C**). Result obtained were opposite to the general mTORC1-induced increase of endocytic activity [4]. To determine the underlying reason for the reduced albumin uptake in S4577D, cell surface expression and intracellular distribution were analyzed by immunohistochemistry and super-resolution STED imaging of MMR2 mutants for intracellular endosomal distribution. Pearson correlation coefficient (PCC) of cell surface stained with wheat germ agglutinin (WGA) and megalin revealed a pronounced surface expression of MMR2 S4577, but still with a high expression intracellularly (Fig. 2C). PCC of S4577A showed significant stronger surface expression as S4577D (Fig. 2D). Super-resolution STED imaging and quantification of colocalization of S4577, S4577A and S4577D with endosomal markers clathrin for clathrin vesicles, EEA1 for early endosomes and Rab11 for recycling endosomes were performed and quantified by using PCC revealing a significantly reduced colocalization of S4577A in clathrin vesicles compared to S4577D and no change in early endosomes and recycling endosomes baseline (Figure S2A – D).

### Inhibition of mTORC1-induced megalin S4577 phosphorylation supports trafficking into clathrin vesicles upon albumin uptake

**Fig. 3:**
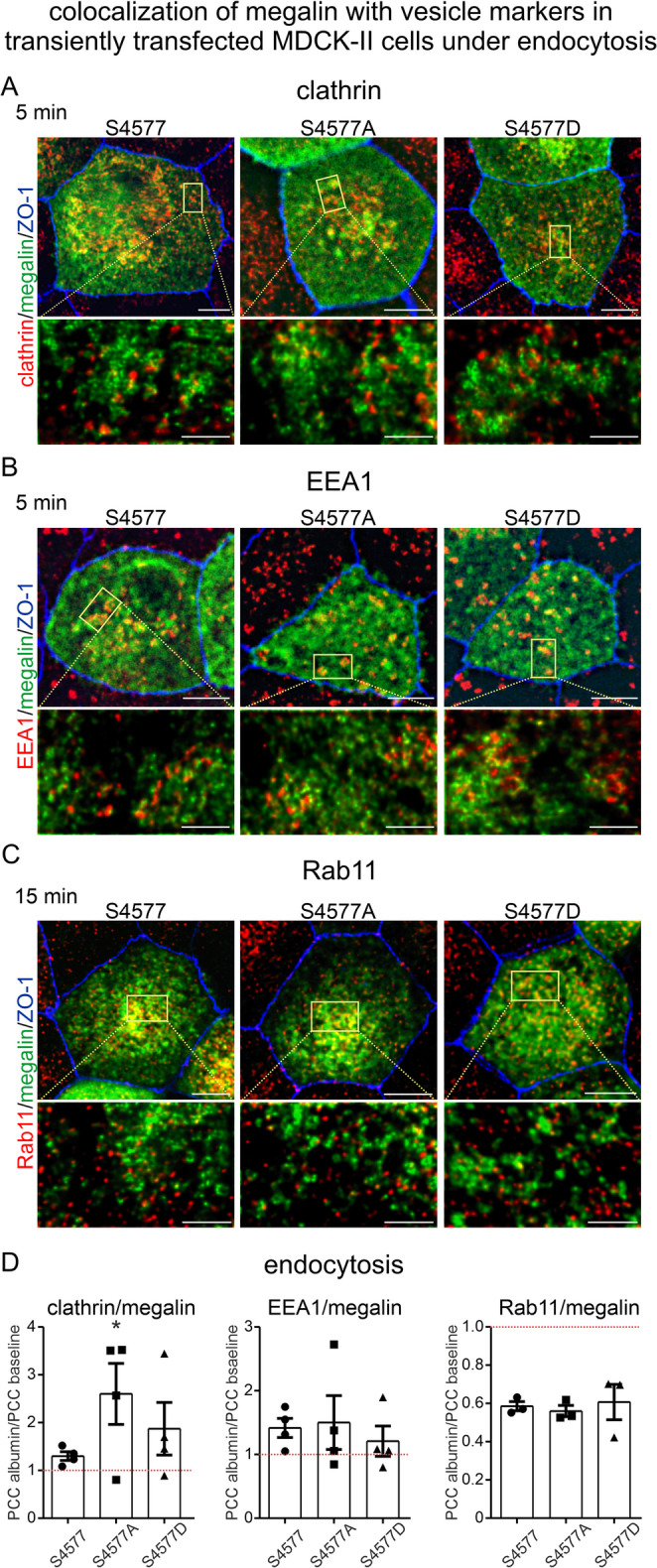
Inhibition of mTORC1-induced S4577 phosphorylation favors trafficking into clathrin vesicles and therefore ligand uptake upon endocytosis. (**A** – **C**) Representative confocal images and higher magnification super-resolution STED images of differentiated MDCK-II cells transiently transfected with megalin MMR2 mutants upon albumin uptake for 5 and 15 min. Cells were triple stained with anti-megalin to identify mutants in green, with endosomal markers clathrin for clathrin vesicles (**A**), EEA1 for early endosomes (**B**) and Rab11 for recycling endosomes in red (**C**), respectively, and with anti-ZO-1 for tight junctions/cell borders in blue to verify cell differentiation. Scale bar = 5 μm and in higher magnification STED image scale bar = 1 μm. (**D**) Quantitative analysis of Pearson correlation coefficient (PCC) of megalin MMR2 mutants with vesicle marker clathrin, or EEA1 or Rab11 upon albumin uptake compared to the respective baseline PCC under starvation condition. Values are mean ± SEM. Approx. 50 cells per *n* = 3–4 independent experiments were evaluated. Mann-Whitney-U test was used to compare means values of PCC in endocytic conditions versus the corresponding baseline value for each megalin MMR2 construct separately, * *P* < 0.05

To analyze the underlying reason for altered albumin uptake in megalin mutants, we determined a possible impact of mTORC1-induced S4577 phosphorylation on megalin trafficking. MDCK-II cells transiently transfected with megalin MMR2 mutants S4577, S4577A and S4577D, respectively, were starved followed by incubation of albumin at 4 °C (pulse) and chased at 37 °C for 5 or 15 min. Double staining of megalin MMR2 mutants with anti-megalin for identification of transfected megalin mutants and with endosomal markers anti-clathrin for clathrin vesicles or anti-EEA1 for early endosomes at 5 min or with anti-Rab11 for recycling endosomes at 15 min was performed (Fig. 3A **– C**). Quantitative colocalization analysis using Pearson correlation coefficient (PCC) of megalin mutants with either clathrin, EEA1 or Rab11 under endocytic conditions were normalized to baseline PCC under starvation condition (Fig. 3D). Upon albumin uptake, megalin MMR2 S4577A mutant showed a significant higher colocalization with clathrin suggesting a stronger involvement of the non-phosphorylated megalin MMR2 mutant in cellular endocytosis. Results were verified in BN16 cells, a differentiated yolk sac cell line with high endogenous megalin expression [13]. Transfection of BN16 cells with MMR2 S4577A mutant lead to a significant higher colocalization with clathrin compared to megalin MMR2 S4577D mutant upon albumin endocytosis (Figure S2E, F), confirming results obtained in MDCK-II cells. In BN16 cells, megalin expression resulting from MMR2 transfection were confirmed to traffic similar as endogenous megalin.

### mTORC1-induced megalin S4577 phosphorylation enhances binding affinity of ARH and alters proteolytic processing of megalin cytoplasmic domain

**Fig. 4:**
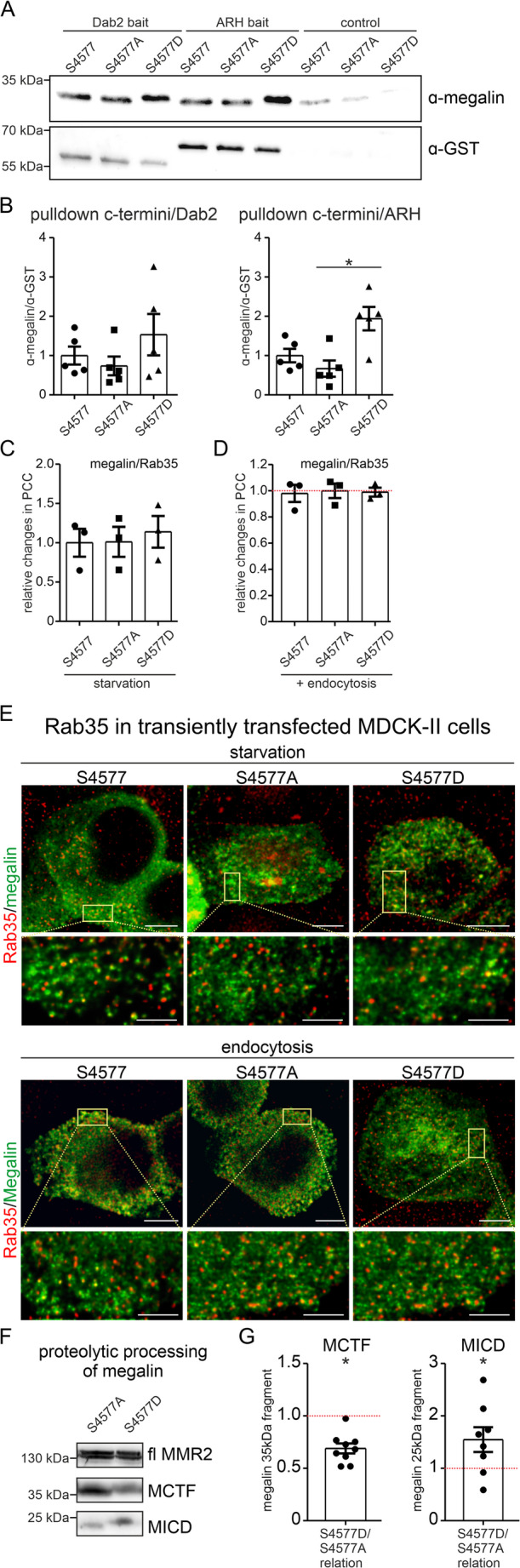
mTORC1-induced megalin S4577 phosphorylation induces binding to ARH and increases proteolytic processing of megalin C-terminus. (**A** and **B**) Representative western blots of pulldown assay using GST-coupled autosomal recessive hypercholesterolemia (ARH) or disabled homolog 2 (Dab2) and megalin C-termini S4577 wildtype, S4577A and S4577D mutants (**A**). Graph of respective densitometric evaluation (**B**). Values are mean ± SEM. *n* = 5 independent experiments were evaluated. Kruskal-Wallis test followed by Dunns post test, * *P* < 0.05. (**C** - **E**) Quantitative colocalization analysis of super-resolution STED images using Pearson correlation coefficient (PCC) between megalin MMR2 mutants and Rab35 obtained from MDCK-II cells upon starvation condition (**C**) or 5 min albumin uptake (**D**) double stained with anti-megalin to identify megalin mutants in green and with Rab35 in red (**E**). Values were normalized to values obtained from S4577 megalin wildtype construct. Scale bar = 5 μm and in higher magnification STED image scale bar = 1 μm. (**F** and **G**) Representative western blots of megalin C-terminus fragments in megalin MMR2 mutants transfected HEK293 cells (**F**) and respective graph of densitometrical evaluation (**G**). fl. MMR2 is full length megalin mini-construct, MCTF is megalin cytoplasmic tail fragment and MICD is soluble megalin intracellular domain. Values are mean ± SEM. *n* = 8 independent experiments were evaluated. Mann-Whitney-U test, * *P* < 0.05

Because of the localization of the mTORC1-induced megalin S4577 phosphorylation site between the two NPxY motifs which are binding sites for the adaptor proteins autosomal recessive hypercholesterolemia (ARH) and disabled homolog 2 (Dab2) (Fig. 1A), we hypothesized that the mTORC1-induced phosphorylation might affect binding affinity between megalin and ARH or Dab2. Pulldown assays using GST-coupled Dab2 or ARH proteins and megalin C-termini S4577 wildtype, S4577A and S4577D mutants revealed a significant higher binding affinity of ARH to the megalin C-terminus mutant S4577D in comparison to the S4577A mutant (Fig. 4A, B). Earlier published data suggested that ARH may guide megalin through the recycling endosomes and thereby preventing fast-recycling route of the scavenger receptor from early endosomes back to plasma membrane via connecdenn2/Rab35-mediated pathway [6]. To determine whether mTORC1-induced megalin S4577 phosphorylation may guide megalin to either the slow or fast recycling route, colocalization of megalin MMR2 mutant with Rab35 in MDCK-II cells was performed. Pearson correlation coefficient (PCC) of megalin MMR2 mutants with Rab35 at baseline under starvation condition or upon albumin uptake for 5 min revealed no significant difference between mutants and conditions suggesting the activation or inhibition of mTORC1-induced phosphorylation does not affect fast recycling pathway of megalin (Fig. 4C - E). Furthermore, upon extracellular domain shedding of megalin, the C-terminal fragment is cleaved by metalloproteases on the cell surface releasing an approx. 35 kDa megalin C-terminal fragment (MCTF) which was shown to be further cleaved by the gamma-secretase into smaller megalin intracellular domain fragments (MICD) within the recycling endosomes compartment in an ARH-dependent manner [6]. Comparing the proteolytic processing of the megalin C-terminal part in HEK293 cells between megalin mutants revealed in MMR2 S4577D in comparison to MMR2 S4577A a significant higher amount of MICD expression due to cleaved MCTF with less MCTF expression level (Fig. 4F, G). These results suggest, that the phosphorylation of megalin by mTORC1 increases the affinity to ARH, transporting megalin into recycling endosomes where proteolytic processing presumably by gamma-secretase occurs.

### mTORC1-induced megalin S4577 phosphorylation supports cell proliferation and megalin is localized to the mitotic spindle pole

**Fig. 5:**
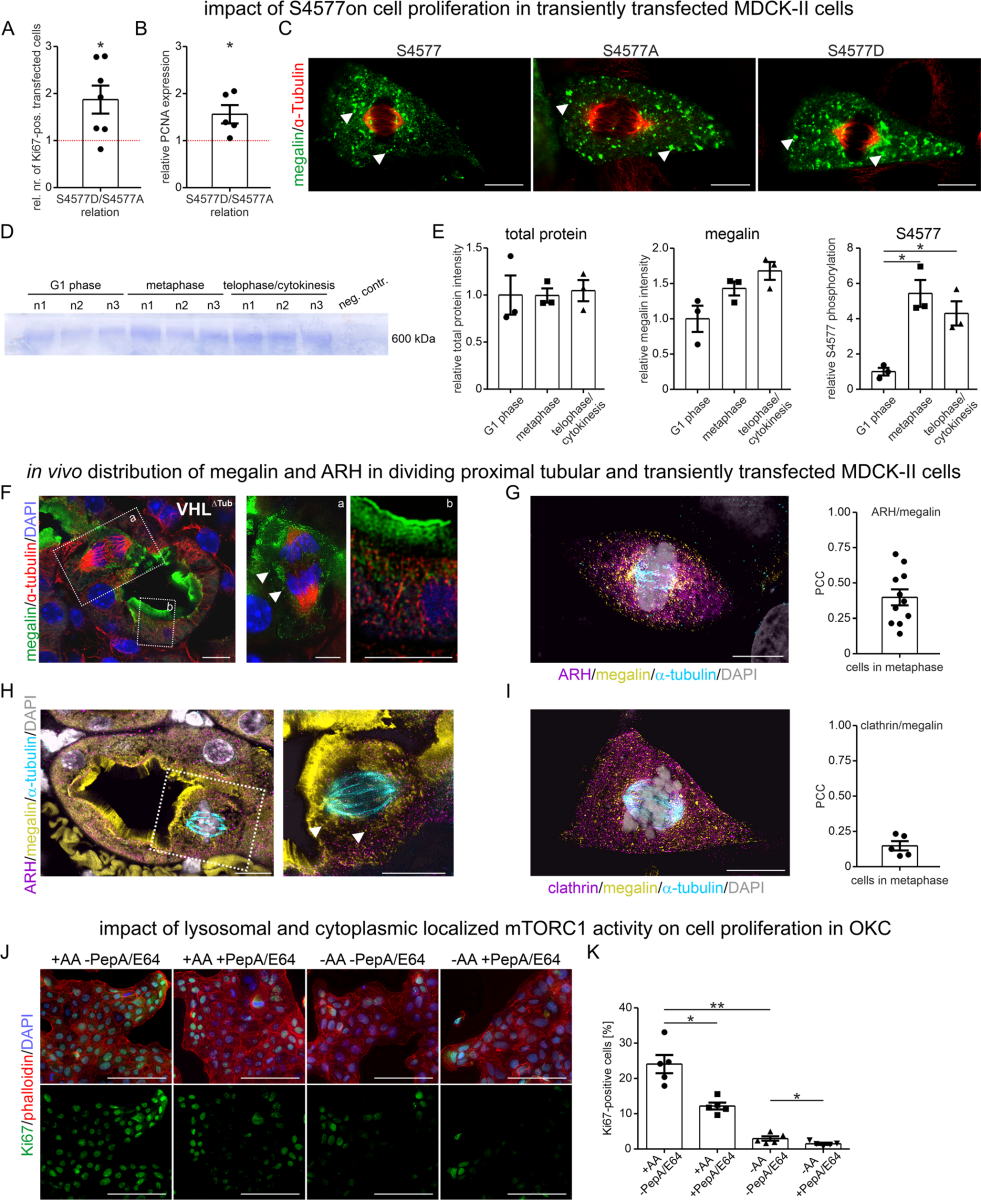
mTORC1-induced megalin S4577 phosphorylation increases cell proliferation and megalin colocalizes with ARH at the spindle pole. (**A**) Graph of analysis determining the number of Ki67-positive cells in transfected megalin MMR2 mutant MDCK-II cells showing a significant higher number of Ki67-positive cells in megalin MMR2 S4577D mutants compared to MMR2 S4577A mutants. Values are mean ± SEM. *n* = 7 independent experiments were evaluated. Mann-Whitney-U test, * *P* < 0.05. (**B**) Graph of densitometrical evaluation of PCNA expression in transfected megalin MMR2 mutant MDCK-II cells showing a significant higher PCNA expression in megalin MMR2 S4577D mutants compared to MMR2 S4577A mutants. Values are mean ± SEM. *n* = 5 independent experiments were evaluated. Mann-Whitney-U test, * *P* < 0.05. (**C**) Representative super-resolution images of transfected megalin MMR2 mutants MDCK-II cells double stained with anti-megalin to identify mutants in green and anti-α-tubulin in red to mark mitotic spindles in cells undergoing mitosis. Arrow heads pointing to megalin protein clusters. Scale bar = 10 μm. (**D**) Coomassie stained SDS-Page of immunoprecipitated megalin at 600 kDa using BN16 cells synchronized to G1 phase, metaphase or telophase/cytokinesis. (**E**) Respective graphs of quantitative mass spectrometry analysis of total protein, megalin protein and S4577 phosphorylated megalin using BN16 cells synchronized to G1 phase, metaphase or telophase/cytokinesis. Values are mean ± SEM. *n* = 3 independent experiments were evaluated. Unpaired t-test, * *P* < 0.05. (**F**) Representative super-resolution image of a proximal tubule profile from *vhl* (von-Hippel-Lindau)-depleted cells triple staining for megalin (green), α-tubulin for mitotic spindles (red) and nuclei with DAPI (blue). Higher magnification images are depicted on the right. Arrow heads point to megalin protein clusters. Scale bar = 5 μm. (**G**) Representative super-resolution image of quadruple staining for ARH (magenta), megalin (yellow), α-tubulin (cyan) for mitotic spindles and nuclei with DAPI (grey) of megalin MMR2 S4577 transiently transfected MDCK-II cells undergoing mitosis. Scale bar = 10 μm. Pearson correlation coefficient (PCC) between megalin and ARH. (**H**) Representative super-resolution image of a proximal tubule profile from *vhl*-depleted cells quadruple staining for ARH (magenta), megalin (yellow), α-tubulin (cyan) for mitotic spindles and nuclei with DAPI (grey). Higher magnification images are depicted on the right. Arrow heads point to megalin protein clusters. Scale bar = 10 μm. (**I**) Representative super-resolution image of quadruple staining for clathrin (magenta), megalin (yellow), α-tubulin (cyan) for mitotic spindles and nuclei with DAPI (grey) of megalin MMR2 S4577 transiently transfected MDCK-II cells undergoing mitosis. Scale bar = 10 μm. Graph of Pearson correlation coefficient (PCC) between megalin and clathrin. (**J** and **K**) Representative images (**J**) and quantification (**K**) of Ki67-positive OKC treated with media containing amino acids (+ AA) for mTORC1 activation; with pepstatin A (PepA) and E-64 for lysosomal mTORC1 inhibition or media without amino acids (-AA) for inhibition of non-lysosomal mTORC1 and stained with Ki67 (green), actin filaments (phalloidin, red) and nuclei with DAPI (blue). Scale bar = 100 μm. Values are mean ± SEM. *n* = 5 independent experiments were evaluated. Kruskal-Wallis test followed by Dunns post test, * *P* < 0.05, ** *P* < 0.01

So far, we found that mTORC1-induced megalin S4577 phosphorylation lowers endocytosis and leads to higher binding affinity to ARH. However, mTORC1 is also known to support mitosis and cell proliferation, which additionally can be ascribed to ARH [23, 24], and which prompted us to analyze the possibility that mTORC1-induced phosphorylation on megalin C-terminus affects cell proliferation and mitosis. First, we assessed cell proliferation by determining the Ki67 and PCNA protein expression of megalin MMR2 mutant expressing MDCK-II cells. Both proteins show nuclear expression in dividing cells and are therefore frequently used as markers of cell proliferation. Megalin MMR2 S4577D mutant showed significantly higher expression of Ki67 and PCNA in comparison to MMR2 S4577A mutant (Fig. 5A, B). These results were confirmed in transiently transfected, endogenously megalin expressing BN16 cells (Figure S3A). We further analyzed the expression of megalin MMR2 mutants in MDCK-II cells during metaphase/early anaphase of mitosis. All megalin MMR2 mutants were colocalized with α-tubulin at the spindle pole (Fig. 5C) without differences in Pearson correlation coefficient between the megalin MMR2 mutants and α-tubulin. In addition, in cells undergoing mitosis, expression pattern of megalin was altered and megalin appeared to be stored in large protein clusters (Fig. 5C, arrow heads). To analyze the physiological relevance of this megalin S4577 phosphorylation site, we determined the amount of endogenous megalin S4577 phosphorylation during the cell cycle in BN16 cells. In comparison to the G1 phase, a significant higher amount of megalin S4577 phosphorylation occurred in metaphase and late telophase/cytokinesis (Fig. 5D, E). We identified another megalin phosphorylation site S4464, which remained unaltered and unknown to date (Figure S3B). To determine whether megalin is localized to the spindle apparatus in vivo during metaphase, transgenic mice with targeted renal epithelial deletion of *von-Hippel Lindau* (*vhl*) with high cellular epithelial proliferation rate [8] were used and demonstrated in super-resolution STED imaging analysis a colocalization of megalin with α-tubulin at the spindle pole similarly to our observation in megalin MMR2 mutant transfected MDCK-II cells (Fig. 5F, Figure S3C). We also observed in vivo altered megalin expression as protein clusters (Fig. 5F arrow heads). To determine whether megalin supports ARH function in MDCK-II cells undergoing mitosis, super-resolution STED imaging colocalization analysis of megalin MMR2 with ARH were performed. In cells during metaphase, a high Pearson correlation coefficient (PCC) was found (Fig. 5G, Figure S3D). Further quadruple staining of ARH, megalin, α-tubulin and nuclei in vivo revealed colocalization of ARH with megalin and α-tubulin at the spindle pole (Fig. 5H, Figure S3E). Note, protein clusters of megalin (Fig. 5H, arrow heads). For comparison we determined also the PCC for megalin and clathrin, as another endocytic protein involved in cell cycle progression. Megalin was found to be colocalized with clathrin albeit to much lesser extent compared to ARH (Fig. 5I, Figure S3F).

Recently, mTORC1 downstream function was nicely presented to be spatially separated into a distinct lysosomal and non-lysosomal pool phosphorylating different substrates in response to various nutrients and amino acid sources [20, 25]. To determine which mTORC1 pool may regulate cell proliferation, we incubated the established proximal tubule cells line, opossum kidney cells (OKC), with either normal OKC media (containing amino acids, but lacking FCS), OKC media supplemented with inhibitors of lysosomal hydrolases pepstatin A (PepA) and E64, with starvation media (without amino acids and FCS) or with starvation media supplemented with PepA and E64 for 24 h. Cell proliferation as determined by nuclear Ki67 expression revealed that blocking lysosomal amino acid supply significantly reduced cell proliferation (Fig. 5J and K). In comparison, total amino acid deprivation reduced cell proliferation even stronger to about one tenth and could further be diminished by additional lysosomal blockade of its hydrolases. As shown previously, amino acid deprivation strongly reduced the phosphorylation of protein S6 and of transcription factor EB (TFEB), belonging respectively to the downstream target of the cytoplasmic or the lysosomal mTORC1 pool (Figure S3G and S3H). In contrast, blockade of lysosomal hydrolases by PepA and E64 over 24 h did not affect phosphorylation of TFEB, which may be due to the long-term treatment necessary to determine cell proliferation.

### Megalin affects cell proliferation and supports redistribution of ARH and recycling endosomes to intercellular bridges

**Fig. 6:**
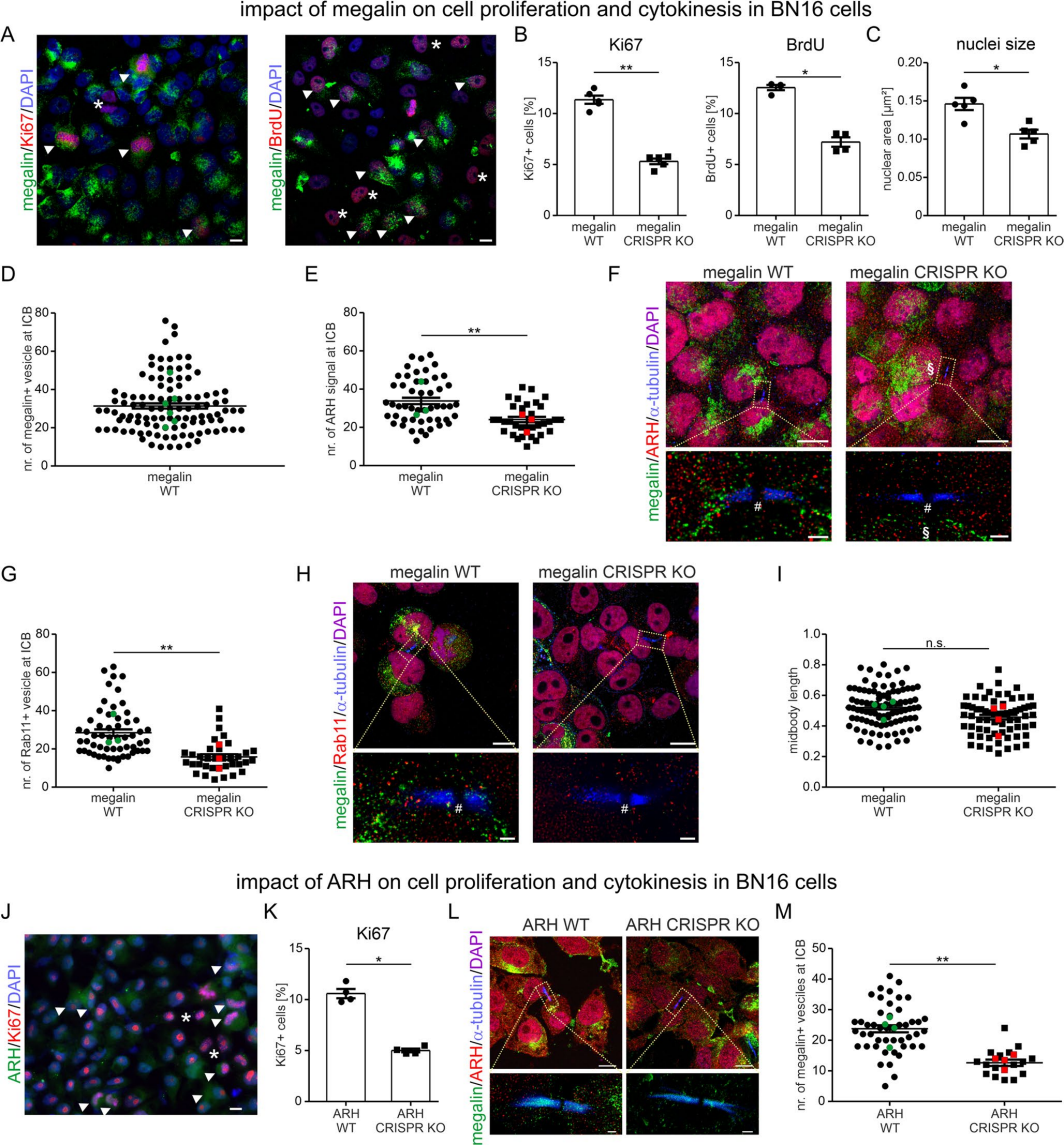
Megalin supports cell proliferation and assists for redistribution of ARH and recycling endosomes to intercellular bridges. (**A** and **B**) Representative images of Ki67- or BrdU-stained BN16 mixed wildtype and megalin-deficient cells (**A**). Ki67 or BrdU in red, megalin in green and nuclei in blue. Scale bar = 10 μm. Arrow heads point to megalin wildtype cells and asterisks mark the megalin-deficient cells. Graph of respective evaluations (**B**). Values are mean ± SEM. *n* = 4–5 independent experiments were evaluated. Mann-Whitney-U test, * *P* < 0.05, ** *P* < 0.01. (**C**) Graph of results obtained from the evaluation of the nuclear size. Values are mean ± SEM. *n* = 5 independent experiments were evaluated. Mann-Whitney-U test, * *P* < 0.05. (**D**) Graph of number of megalin-positive vesicles at the pole of the intercellular bridge (ICB) in BN16 wildtype cells. Individual ICBs are presented in black dots, means of independent experiments (*n* = 6) in green. (**E**) Graph of number of ARH signals at the ICB pole in BN16 wildtype and megalin-deficient cells. Individual ICBs are presented in black dots, means of independent experiments (*n* = 3) in green for megalin wildtype cells and in red for megalin-deficient cells. Mann-Whitney-U test, ** *P* < 0.01. (**F**) Representative confocal and super-resolution STED image (below) of quadruple-stained mixture of BN16 wildtype and megalin-deficient cells of megalin in green, ARH in red, α-tubulin in blue and nuclei in magenta. Scale bar = 10 μm and in higher magnification STED image scale bar = 1 μm. Section character marks a megalin-expression neighboring cell and the hash marks midbodies. (**G**) Graph of number of Rab11-positive vesicles at the ICB pole in BN16 wildtype and megalin-deficient cells. Individual ICBs are presented in black dots, means of independent experiments (*n* = 3) in green for megalin wildtype cells and in red for megalin-deficient cells. Mann-Whitney-U test, ** *P* < 0.01. (**H**) Representative confocal and super-resolution image (below) of quadruple-stained mixture of BN16 wildtype and megalin-deficient cells of megalin in green, Rab11 in red, α-tubulin in blue and nuclei in magenta. The hash marks midbodies. Scale bar = 10 μm and in higher magnification STED image scale bar = 1 μm. (**I**) Graph of midbody length in BN16 wildtype and megalin-deficient cells during cytokinesis. Individual midbodies are presented in black dots, means of independent experiments (*n* = 4) in green for megalin wildtype cells and in red for megalin-deficient cells. (**J**) Representative images of Ki67-stained BN16 mixed wildtype and ARH-deficient cells. Ki67 in red, ARH in green and nuclei in blue. Scale bar = 10 μm. Arrow heads point to ARH wildtype cells and asterisks mark the ARH-deficient cells. (**K**) Evaluation of Ki67-positive cells in BN16 wildtype and ARH-deficient cells. Values are mean ± SEM. *n* = 4 independent experiments were evaluated. Mann-Whitney-U test, *P < 0.05. (**L**) Representative confocal and super-resolution STED image (below) of quadruple-stained mixture of BN16 wildtype and ARH-deficient cells of megalin in green, ARH in red, α-tubulin in blue and nuclei in magenta. Scale bar = 10 μm and in higher magnification STED image scale bar = 1 μm. (**M**) Graph of number of megalin-positive vesicles at the ICB pole in BN16 wildtype and ARH-deficient cells. Individual ICBs are presented in black dots, means of independent experiments (*n* = 4) in green for ARH wildtype cells and in red for ARH-deficient cells. Mann-Whitney-U test, ** *P* < 0.01

To analyze in more detail whether megalin is involved in cell proliferation and division, we generated megalin-deficient BN16 cells. In comparison to wildtype cells, megalin-deficient cells were significantly less positive for Ki67and less for BrdU, a substance incorporating in dividing cells (Fig. 6A and **B**). In addition, smaller nuclei size was determined in megalin-deficient cells in comparison to respective wildtype cells as well, suggesting that megalin is also involved in post-mitotic events for proper nuclear morphology (Fig. 6C). During mitosis, ARH was shown to localize to and to be important for mitotic spindle formation [24]. Therefore, we question whether megalin may also affect spindle formation. In comparison to wildtype cells, megalin-deficient cells demonstrated no alteration in colocalization of ARH to g-tubulin-stained mitotic spindles, no difference in ARH signal intensity at the spindle pole and also no difference in the distance between spindle poles were observed (Figure S4A - D), suggesting that megalin may not affect ARH function during metaphase. ARH was further shown to be involved in cytokinesis [26], therefore we first assed a localization of megalin in close proximity to the intercellular bridges (ICB) and indeed megalin-positive vesicles are encountered at the ICB pole during cytokinesis (Fig. 6D). In addition, in comparison to wildtype cells, megalin-deficient cells showed significantly less ARH signals (Fig. 6E, F) and less Rab11-positive vesicles (Fig. 6G, H) at the ICB pole. We therefore hypothesize, that megalin transports ARH and Rab11-positive vesicles to the cleavage furrow presumably necessary for membrane abscission and terminal scission step in late cytokinesis [27]. Midbody length however, remained unaltered (Fig. 6I).

### ARH affects cell proliferation and megalin distribution on intercellular bridges

To confirm previous results of ARH on cell proliferation in endothelial cells [24], we generated ARH-deficient BN16 cells and determined cell proliferation and the number of megalin-positive vesicles on ICB. As observed in megalin-deficient cells, knockout of ARH lead to a significant reduction of proliferating Ki67-positive cells in comparison to the wildtype cells (Fig. 6J, K) and showed a reduced number of megalin-positive vesicles at the ICB pole during cytokinesis (Fig. 6L, M). In addition, megalin expression was significantly reduced in differentiated BN16 cells (Figure S4E) as well.

**Fig. 7:**
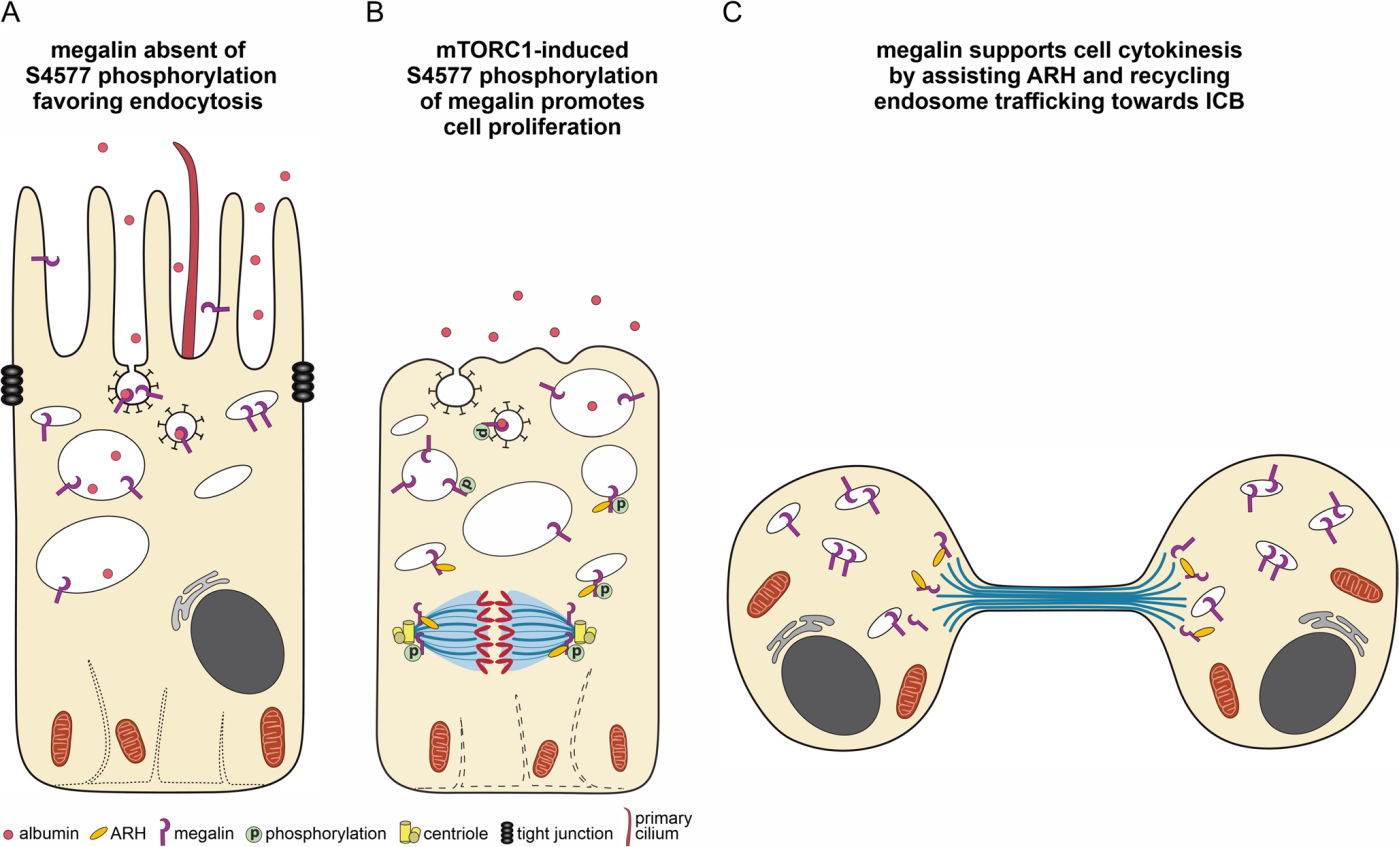
Summary schemes. (**A**) Wildtype megalin absent of S4577 phosphorylation favoring cell growth and endocytosis. (**B**) mTORC1-induced megalin phosphorylation at S4577 supports cell proliferation. Megalin colocalizes with ARH at the spindle pole in metaphase to assist ARH function. (**C**) During cytokinesis megalin supports ARH and Rab11-positive vesicles trafficking towards the ICB pole, both necessary for cell abscission

From our results, we summarize that absence of mTORC1-mediated phosphorylation of megalin S4677 site favors cell growth and clathrin-mediated endocytosis (Fig. 7A). mTORC1-mediated megalin phosphorylation at S4577 however, increases the affinity to ARH and augments cell proliferation (Fig. 7B). During cytokinesis, megalin, ARH and Rab11-positive recycling endosomes can be encountered at the ICB pole and are a prerequisite necessary for proper cell division in late cytokinesis most probably by ensuring membrane transport (Fig. 7C).

## Discussion

In our study, we observed that mTORC1-induced phosphorylation of megalin cytoplasmic domain at S4577 reduced the endocytosis rate and induces a redistribution into clathrin vesicles. This phosphorylation site is located nearby the NPxY motifs of megalin cytoplasmic domain and we further found an increased binding affinity of megalin to ARH and consecutive proteolytic processing of megalin C-terminal part to generate the MICD fragment. In addition, this higher binding affinity to ARH supports the ARH function in cell cycle progression. mTORC1-induced megalin S4577 phosphorylation is altered during the cell cycle with highest expression in metaphase and telophase/cytokinesis demonstrating the physiological importance. During cytokinesis, megalin ensures the amount of recycling endosomes and ARH at the cleavage furrow important for cell division. These results show for the first time the involvement of megalin in cell cycle progression and the importance of mTORC1 signaling enabling appropriate cell division.

### mTORC1-mediated phosphorylation of megalin cytoplasmic domain at S4577 reduces endocytosis rate and increases interaction with ARH

Our results of pulse-chase endocytosis assays revealed that the mTORC1-induced phosphorylation of megalin at S4577 significantly reduced the amount of endocytosed albumin. At the first glance, these finding may be contrary to earlier observations of our group, where devoid of mTORC1 signaling by genetic deletion of *Raptor* led to an overall decrease in endocytosis. As underlying mechanism reduced phosphorylation of Arf GTPase guanine nucleotide exchange factor GBF-1 and lower abundance of sorting nexin-8 and Rab10, proteins involved endosomal vesicles trafficking, were identified [4, 28]. This discrepancy may be explained that the majority of proximal tubule cells, as slow cycling cells, are quiescent to enable normal proximal tubule function including endocytosis. Only a minority of the proximal tubule cells undergo cell division (approx. 0.007% [14]),, where mTORC1 has a specific role in cell cycle progression by phosphorylating megalin at S4577 as discussed below and this may explain the long-term effect of Raptor-depleted mice, the cortex reduction and could solve the discrepancy.

To alter cellular endocytosis rate, megalin was shown to traffic either through a fast-recycling pathway from early endosome back to the plasma membrane or enter the slow recycling pathway via the endocytic recycling compartment by ARH [6]. The mTORC1-induced megalin phosphorylation site S4577 is located nearby the NPxY motifs for ARH and Dab2 binding, therefore we hypothesized that the binding affinity of megalin to its adaptor proteins ARH and Dab2 may be affected by the phosphorylation. Indeed, pulldown analysis of ARH, Dab2 and megalin mutants revealed that the phosphorylation of S4577 on megalin C-terminal domain led to an increased ARH binding in comparison to absent mTORC1-induced megalin phosphorylation at S4577. The Dab2 binding affinity to megalin remained unaffected. To confirm that mTORC1-induced phosphorylation on megalin C-terminal domain initiates the slow recycling pathway of megalin colocalization studies of megalin mutants were performed with Rab35 for Rab35-positive vesicles of the fast-recycling pathway. Surprisingly, no alteration of colocalization between megalin mutants and Rab35 were observed, suggesting that binding affinity of megalin with S4577 phosphorylation and ARH is independent of the pathway used (slow or fast-recycling pathway). However, the binding of megalin to ARH was also established as a prerequisite for intramembrane proteolysis and consecutive suppression of transcription [6, 29]. This two-step intramembrane proteolysis comprises first a cleavage at the cell surface by a matrix metalloprotease resulting in a membrane-bound megalin fragment (MCTF [30, 31]),, which is then internalized into the endosomal system and in a second step further processed by gamma-secretase resulting in a small soluble megalin fragment (MICD), which is able to translocate into the nucleus and suppress the transcription of megalin and sodium hydrogen exchanger 3 [6, 28, 29]. Indeed, upon inhibition of proteasomal degradation mTORC1-induced megalin S4577D showed a significant higher abundance of MICD in comparison to unphosphorylated megalin S4677A corroborating our findings of ARH binding affinity. Unfortunately, using conventional microscopy we were unable to detect nuclear expression of MICD. As shown before, the suppression of megalin and NHE3 transcription fit with the observation that this phosphorylation helps cells to proliferate and cell division is a cellular state where transcriptional activity is not needed and therefore reduced.

#### Megalin supports ARH function in cell division

Since mTORC1-dependent signaling and ARH were shown to have a common role in cell cycle progression [26, 32], we determined cell proliferation and localization during mitosis. mTORC1-induced phosphorylation of megalin at S4577 significantly increased cell proliferation in comparison to absent mTORC1-dependent phosphorylation. In cells undergoing mitosis, we could localize megalin to the spindle poles which was independent of mTORC1-induced phosphorylation on S4577. In addition, using mice with targeted deletion of renal epithelial *von-Hippel-Lindau* displaying strongly induced epithelial cell proliferation [8, 33], we could also localize megalin to the spindle poles in vivo. Similarly, ARH redistributes in a cell cycle-dependent manner to centrosomes and components of the spindle apparatus, much alike dynein [26]. Clathrin is another endocytosis-associated protein interacting with megalin and described to be involved in cell mitosis by stabilizing fibers of the mitotic spindle [34, 35]. To obtain more information on the role of megalin in mitosis, we performed super-resolution microscopy colocalization studies of megalin with ARH and megalin with clathrin in cells undergoing mitosis where we observed higher colocalization of megalin with ARH compared to megalin with clathrin, suggesting a role for megalin to support ARH function in cellular mitosis. ARH was also shown to participate in cytokinesis [26], which is the physical separation of the two daughter cells and is an integral part of cell division. For successful cytokinesis, formation and constriction of the acto-myosin ring and delivery of new membrane to the cleavage furrow are mandatory [27]. We could observe megalin, ARH and Rab11-positive recycling endosomes at the ICB pole during cytokinesis. Megalin-depletion led to reduced number of Rab11-positive recycling endosomes at the ICB. As demonstrated earlier and by us, depletion of ARH similarly to depletion of Rab11 resulted in reduced proliferation due to defective cytokinesis with formation of binuclear cells [26, 27]. Similarly, in kidney proximal tubule cells, megalin depletion also resulted in a reduced number of recycling endosomes [36] confirming a role for megalin in recycling endosome formation and distribution.

The involvement of mTORC1 signaling in cell cycle progression has been nicely determined recently [37]. mTORC1 and its various downstream targets and functions can be spatially separated intracellularly in a lysosomal and non-lysosomal mTORC1 pool. Both, lysosomal and non-lysosomal nutrient supply affect cell proliferation albeit to different extent. Lysosomal mTORC1 signaling however, remained unaltered due to long-term treatment. mTORC1 activity was presented to vary during the cell cycle and was shown to regulate G2/M checkpoint recovery and to play a role in mitotic spindle formation and subsequent chromosome segregation [38]. mTORC1 signaling on megalin may be another mean by which mTORC1 signaling supports cell cycle progression. We could demonstrate now, that this mTORC1-induced megalin phosphorylation alters during cell cycle and shows highest levels during metaphase and telophase/cytokinesis. mTORC1 signaling may most probably involve other endocytic proteins which need to be examined in future. Along the way, we observed an alteration in megalin intracellular distribution when cells undergoing mitosis. Megalin is then stored in large intracellular vesicles appearing as clusters, irrespective of S4577 phosphorylation. The observed clusters of megalin did not colocalize with clathrin. This phenomenon can be attributed to other membrane receptors involved in clathrin-dependent endocytosis e.g. transferrin receptor or Celsr1, which were endocytosed during mitosis, distributed into condensates, protein clusters, ensuring regulated distribution of receptors to the daughter cells during mitosis [37, 39].

*Weakness of the study.* The proximal tubule like MDCK-II cell line has extremely low megalin expression which has been used in our study to evaluate the differences between the megalin mutants. Although MDCK-II cells perform low endocytosis and are usually not used for endocytosis analysis, upon transient transfection and differentiation for at least 72 h, we observed that the cells were polarized and differentiated as judged by the expression of tight junctions. At that time point a strong uptake of AlexaFluor555-labeled albumin and redistribution of megalin mutants between endocytic compartments was always observed making the cell line suitable for comparative analysis. Although megalin mini-mutants are frequently used to analyze megalin function [7, 16], they still may behave differently to endogenous megalin, which we cannot rule out here. Another weakness of the study is the trafficking behavior of endocytic receptors in MDCK-II cells. Polarization and cell differentiation were shown to be extremely important in the regulation of endocytic trafficking [40]. It has previously been documented that the organization of the recycling pathway and the endocytic and recycling rates of the mini-megalin construct in MDCK-II cells is different from proximal tubule cells endogenously expressing megalin [41, 42]. By generating megalin mutant mice for the S4577 mTORC1-induced phosphorylation site would help to clarify the role of mTORC1 and megalin in cell cycle in vivo. Another weakness of the study is the inability to differentiate in megalin-depleted cells between effects of nutrient deprivation and megalin depletion itself. Further experiments need to be performed to rule out the function of megalin in cell proliferation.

## Conclusion

For the first time, we describe that mTORC1-induced phosphorylation on megalin C-terminal domain acts as a switch on megalin between a cellular involvement in endocytosis or mitosis and cytokinesis. The underlying mechanistic insights should be further investigated in detail in future. mTORC1 signaling pathway could act also on other endocytic proteins involved in cell cycle progression to switch the cell between interphase and mitosis and therefore between cell growth and cell division.

## Supplementary Information

Below is the link to the electronic supplementary material.

Supplementary figure 1 (PNG 777 KB)

Supplementary Material 1**Figure S1:mTORC1-induced S4577 phosphorylation reduces albumin uptake in HK2 cells. **(**A**) Correlation analysis of megalin expression (relative fluorescence intensity) in transfected MDCK-II cells and albumin uptake (relative fluorescence intensity) using two-tailed correlation analysis of Pearson showing a significant correlation between the two parameters. (**B**and**C**) Quantification and representative images depicting albumin uptake of megalin mutants in transiently transfected HK2 cells. Representative images of albumin uptake are shown in green. Cells were co-stained with anti-myc (red) for identification of mutant transfected cells and with phalloidin (grey) to mark cell borders. Nuclei were counterstained with DAPI (blue). Scale bar = 10 µm. Values are mean± SEM of n = 6 individual experiments. Mann-Whitney-U test, *P< 0.05 (TIF 53.5 MB)

Supplementary figure 2 (PNG 635 KB)

Supplementary Material 2 **Figure S2:Baseline megalin distribution is minorly changed upon mTORC1-induced S4577 phosphorylation. **(**A**– **C**) Representative confocal images and higher magnification super-resolution STED images of differentiated MDCK-II cells transiently transfected with megalin MMR2 mutants under starvation conditions. Cells were triple stained with anti-megalin to identify mutants in green, with endosomal markers clathrin for clathrin vesicles (**A**), EEA1 for early endosomes (**B**) and Rab11 for recycling endosomes in red (**C**), respectively, and with anti-ZO-1 for tight junctions/cell borders in blue to verify cell differentiation. Scale bar = 5 µm and in higher magnification STED image scale bar = 1 µm. (**D**) Quantitative analysis of Pearson correlation coefficient (PCC) of megalin MMR2 mutants with vesicle marker clathrin, or EEA1 or Rab11 under starvation condition. Values are mean ± SEM. Approx. 50 cells per n = 3 – 4 independent experiments were evaluated. Kruskal-Wallis test followed by Dunns post test, ** *P*< 0.01. (**E**and** F**) Representative confocal images and higher magnification super-resolution STED images of BN16 cells transiently transfected with megalin MMR2 mutants under starvation conditions and 5 min albumin endocytosis (**E**). Cells were double stained with anti-megalin to identify mutants in green and with anti-clathrin to identify clathrin vesicles. Scale bar= 5 µm and in higher magnification STED image scale bar = 1 µm. Quantitative analysis of Pearson correlation coefficient (PCC) of megalin MMR2 mutants with vesicle marker clathrin upon albumin uptake compared to the respective baseline PCC under starvation condition (**F**). Values are mean ± SEM. Approx. 40 cells per n = 3 independent experiments were evaluated. Mann-Whitney-U test was used to compare PCC values in endocytic conditions versus the corresponding baseline value for each megalin MMR2 construct separately, * *P*< 0.05 (TIF 19.3 MB)

Supplementary figure 3 (PNG 2.56 MB)

Supplementary Material 3**Figure S3. Megalin phosphorylation influences cell proliferation and megalin colocalizes with ARH and minorly with clathrin at the spindle poles in mitotic cells.** (**A**) Graph of analysis determining the number of Ki67-positive cells in transfected megalin MMR2 mutants in BN16 cells showing a significant higher number of Ki67-positive cells in megalin MMR2 S4577D mutants compared to MMR2 S4577A mutants. Values are mean ± SEM. n = 4 independent experiments were evaluated. Mann-Whitney-U test, * *P*< 0.05. (**B**) Respective graph of quantitative mass spectrometry analysis of S4464 phosphorylated megalin using BN16 cells synchronized to G1 phase, metaphase or telophase/cytokinesis. Values are mean ± SEM. n = 3 independent experiments were evaluated. Unpaired t-test. (**C**) Single channel presentation of merge images shown in Figure **5F** for better visualization of megalin distribution in (a) mitotic cells compared to (b) differentiated proximal tubular cells Super-resolution image of a proximal tubule profile from *vhl* (von-Hippel-Lindau)-depleted cells stained for megalin (green) and α-tubulin for mitotic spindles (red). Higher magnification images are depicted on the right. Scale bar = 5 µm. (**D**) Single channel presentation of merge images shown in Figure **5G** for better visualization of ARH and megalin distribution pattern and cluster building at the position of spindle poles. Super-resolution image of quadruple staining for ARH (magenta), megalin (yellow), α-tubulin (cyan) for mitotic spindles and nuclei with DAPI (grey) of megalin MMR2 S4577 transiently transfected MDCK-II cells undergoing mitosis. Scale bar = 10 µm**.**(**E**) Single channel presentation of merge images shown in Figure **5H** for better visualization of ARH and megalin distribution pattern and cluster building at the position of spindle poles* in vivo*. Super-resolution image of a proximal tubule profile from *vhl* (von-Hippel-Lindau)-depleted cells quadruple staining for ARH (magenta), megalin (yellow), α-tubulin (cyan) for mitotic spindles and nuclei with DAPI (grey). Higher magnification images are depicted below. Scale bar = 10 µm. (**F**) Single channel presentation of merge images shown in Figure **5I** for better visualization of clathrin and megalin distribution pattern and cluster building at the position of mitotic spindles and spindle poles. Super-resolution image of quadruple staining for clathrin (magenta), megalin (yellow), α-tubulin (cyan) for mitotic spindles and nuclei with DAPI (grey) of megalin MMR2 S4577 transiently transfected MDCK-II cells undergoing mitosis. Scale bar = 10 µm**.**(**G**and** H**)Densitometrical evaluation (**G**) and representative western blots (**H**) from phosphorylated protein S6, protein S6, phosphorylated transcription factor EB (TFEB), and TFEB using OKC treated with either normal OKC media, OKC media supplemented with pepstatin A (PepA) and E64, with starvation media or with starvation media supplemented with PepA and E64 for 24 hours. Values are mean ± SEM, n = 6 independent experiments were evaluated. Kruskal-Wallis test followed by Dunns post test, * P < 0.05 (TIF 70.9 MB)

Supplementary figure 4 (PNG 2.40 MB)

Supplementary Material 4**Figure S4. Role of megalin during metaphase of mitosis. **(**A**) Quantification of Pearson correlation coefficient (PCC) between of ARH and a-tubulin in BN16 wildtype and megalin-deficient cells in metaphase. Values are mean ± SEM, n = 5 independent experiments were evaluated. (**B**) Quantification of ARH signal intensity at the spindle pole compared to cytoplasmatic ARH signal intensity of BN16 wildtype and megalin-deficient cells in metaphase. Values are mean ± SEM. n = 5 independent experiments were evaluated. (**C**) Super-resolution image of quadruple staining for megalin (yellow), ARH (magenta), α-tubulin (cyan) for mitotic spindles and nuclei DAPI (grey) of BN16 wildtype and megalin-deficient cells in metaphase. Scale bar = 10 µm. (**D**) Quantification of the distance between the spindles poles of BN16 wildtype and megalin-deficient cells in metaphase. Values are mean ± SEM, n = 3 independent experiments were evaluated. (**E**) Quantification of relative fluorescence intensity of megalin expression in wildtype BN16 cells and ARH depleted BN16 cells. Values are mean ± SEM. n = 6 independent experiments were evaluated. Mann-Whitney-U test, * *P*< 0.05 (TIF 100 MB)

Supplementary Material 5 (PDF 4.93 MB)

## Data Availability

The data that support the findings of this study are available in the Materials and Methods, Results, and/or Supplemental Material of this article. The original data concerning the mass spectrometry proteomics data of endogenous megalin in BN16 cells during different cell cycle phases have been deposited to the ProteomeXchange Consortium via the PRIDE [1] partner repository with the dataset identifier PXD071589.
