# Supplementary figures and images for: mTORC1-signaling switches megalin function from endocytosis to cell cycle progression
Source: Cell Mol Life Sci. 2026 May 23;83(1):224. doi: 10.1007/s00018-026-06247-5 (PMC13198606; doi:10.1007/s00018-026-06247-5)

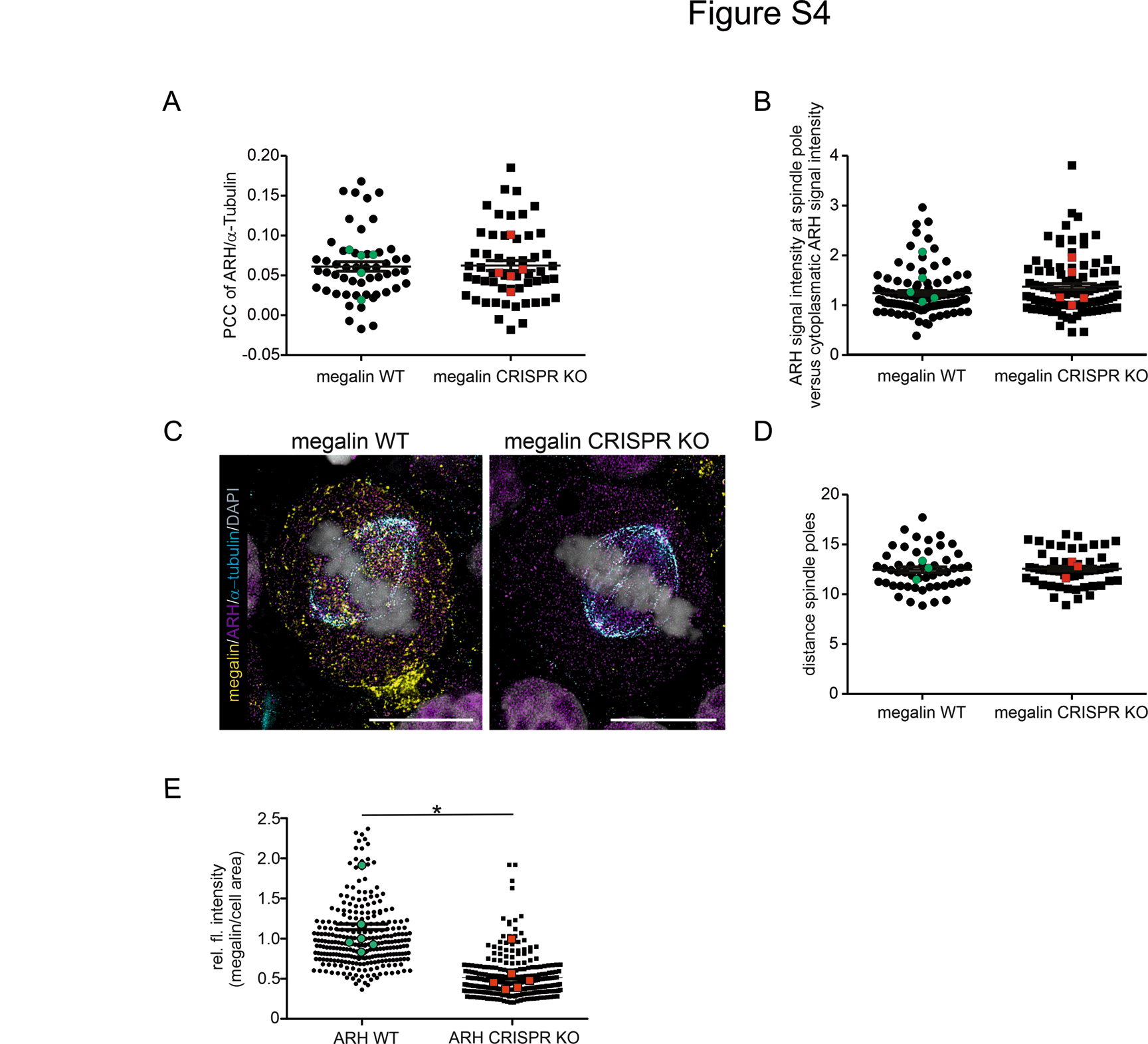

Supplement: Supplementary file 1 — (PNG 777 KB) [file 18_2026_6247_Fig8_ESM.png]

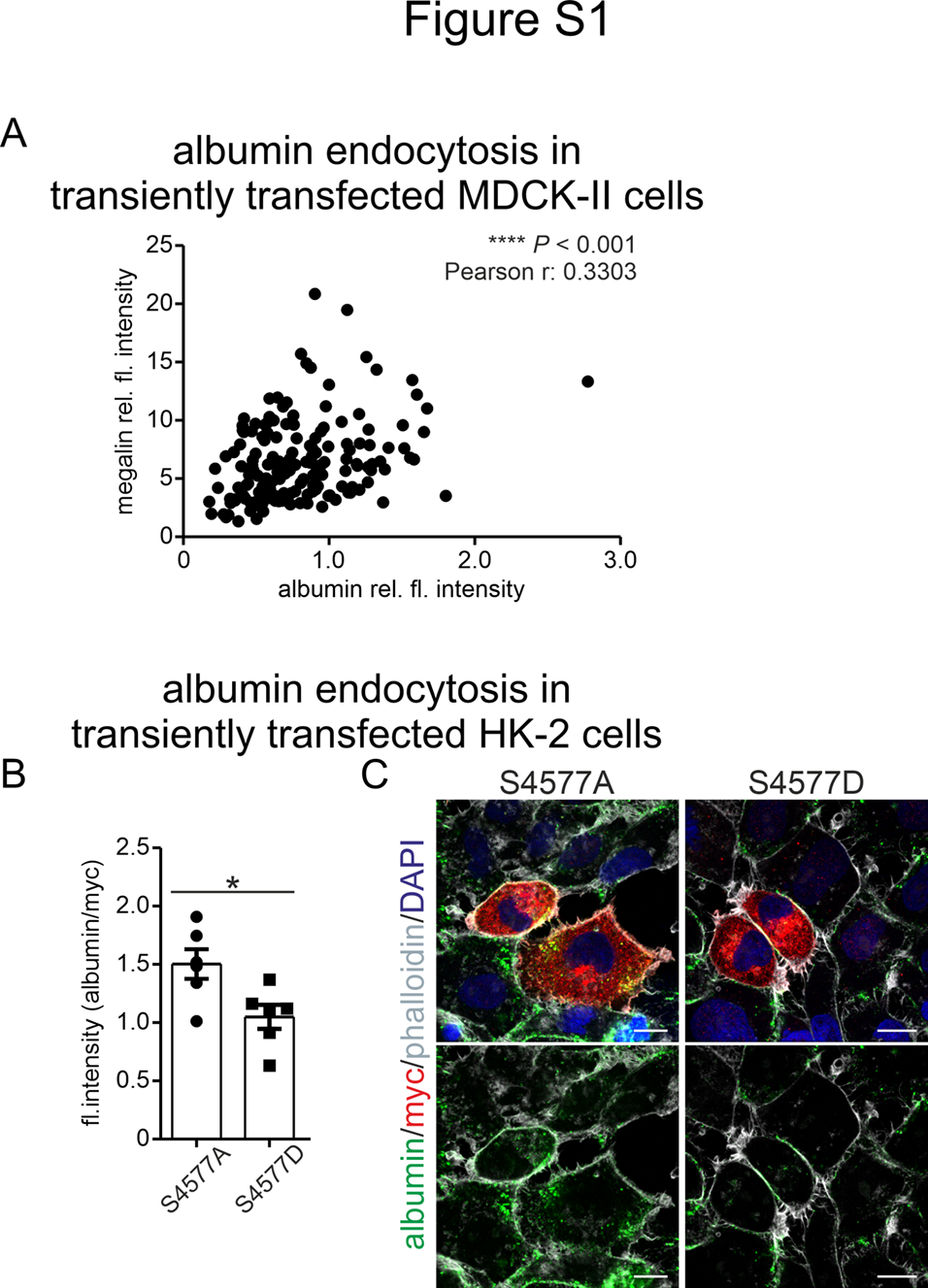

Supplement: Supplementary file 3 — (PNG 635 KB) [file 18_2026_6247_Fig9_ESM.png]

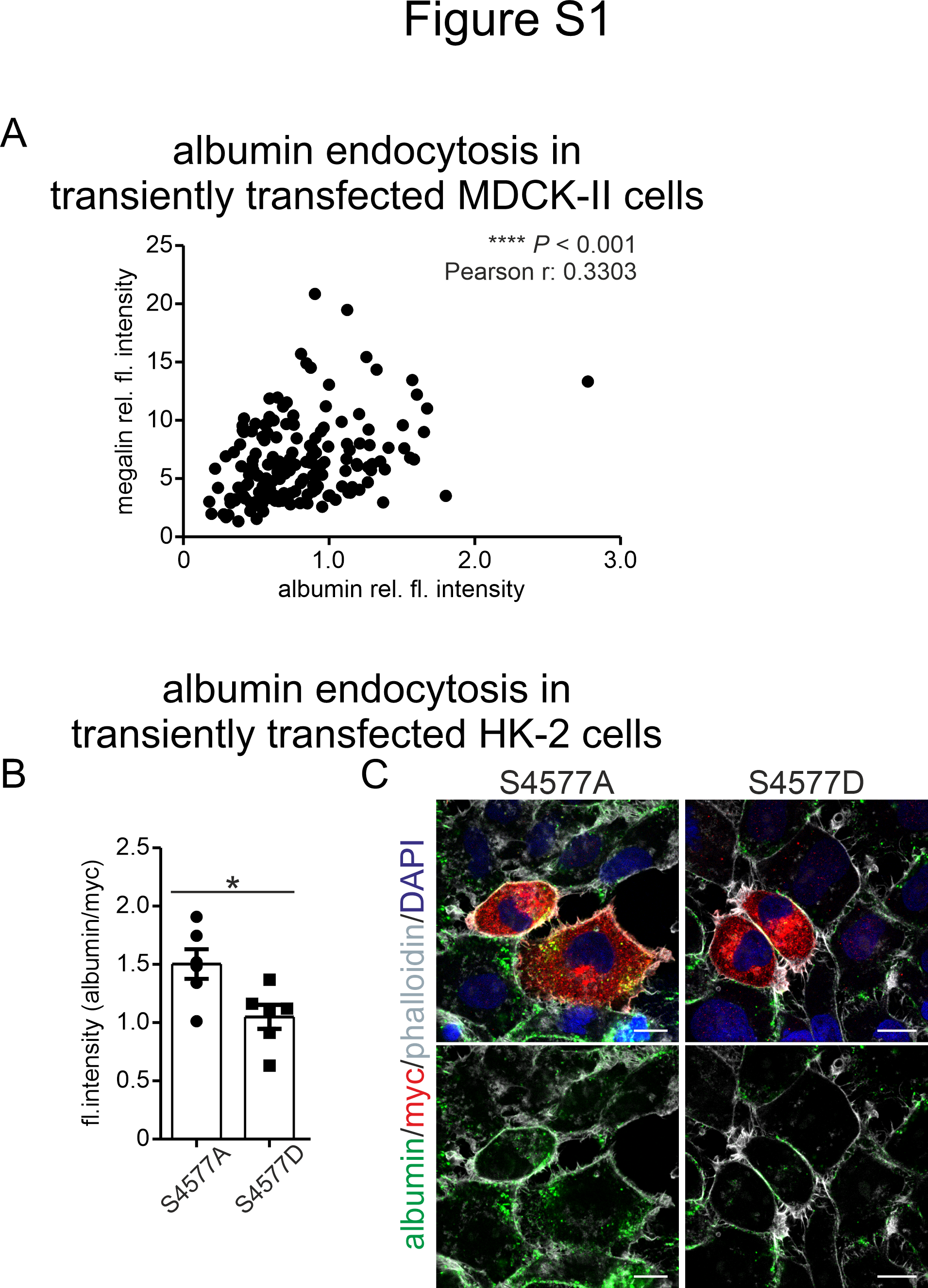

Supplement: Supplementary file 4 — Supplementary Material 2 Figure S2:Baseline megalin distribution is minorly changed upon mTORC1-induced S4577 phosphorylation. (A– C) Representative confocal images and higher magnification super-resolution STED images of differentiated MDCK-II cells transiently transfected with megalin MMR2 mutants under starvation conditions. Cells were triple stained with anti-megalin to identify mutants in green, with endosomal markers clathrin for clathrin vesicles (A), EEA1 for early endosomes (B) and Rab11 for recycling endosomes in red (C), respectively, and with anti-ZO-1 for tight junctions/cell borders in blue to verify cell differentiation. Scale bar = 5 µm and in higher magnification STED image scale bar = 1 µm. (D) Quantitative analysis of Pearson correlation coefficient (PCC) of megalin MMR2 mutants with vesicle marker clathrin, or EEA1 or Rab11 under starvation condition. Values are mean ± SEM. Approx. 50 cells per n = 3 – 4 independent experiments were evaluated. Kruskal-Wallis test followed by Dunns post test, ** P< 0.01. (Eand F) Representative confocal images and higher magnification super-resolution STED images of BN16 cells transiently transfected with megalin MMR2 mutants under starvation conditions and 5 min albumin endocytosis (E). Cells were double stained with anti-megalin to identify mutants in green and with anti-clathrin to identify clathrin vesicles. Scale bar= 5 µm and in higher magnification STED image scale bar = 1 µm. Quantitative analysis of Pearson correlation coefficient (PCC) of megalin MMR2 mutants with vesicle marker clathrin upon albumin uptake compared to the respective baseline PCC under starvation condition (F). Values are mean ± SEM. Approx. 40 cells per n = 3 independent experiments were evaluated. Mann-Whitney-U test was used to compare PCC values in endocytic conditions versus the corresponding baseline value for each megalin MMR2 construct separately, * P< 0.05 (TIF 19.3 MB) [file 18_2026_6247_MOESM2_ESM.tif]

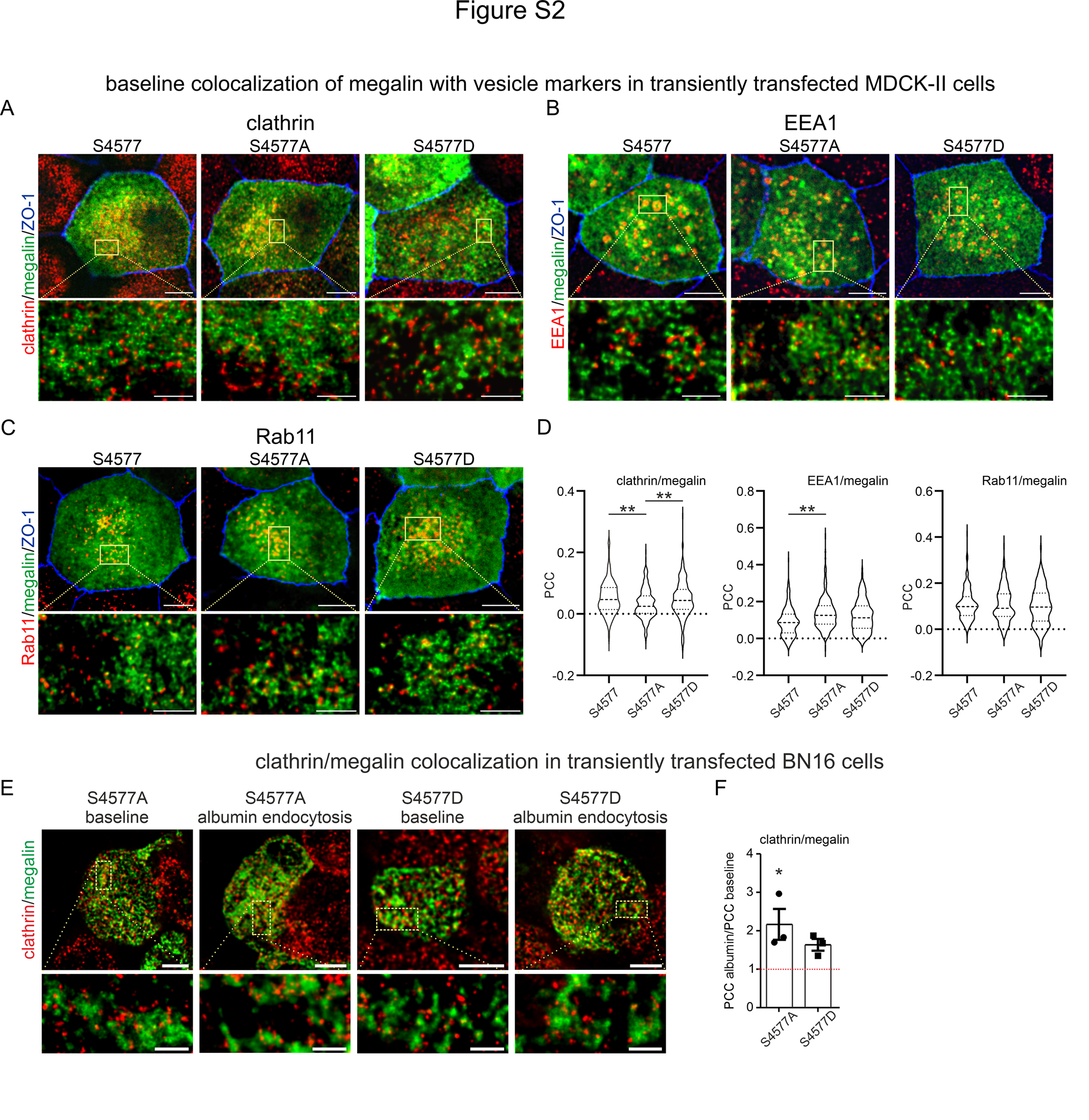

Supplement: Supplementary file 5 — (PNG 2.56 MB) [file 18_2026_6247_Fig10_ESM.png]

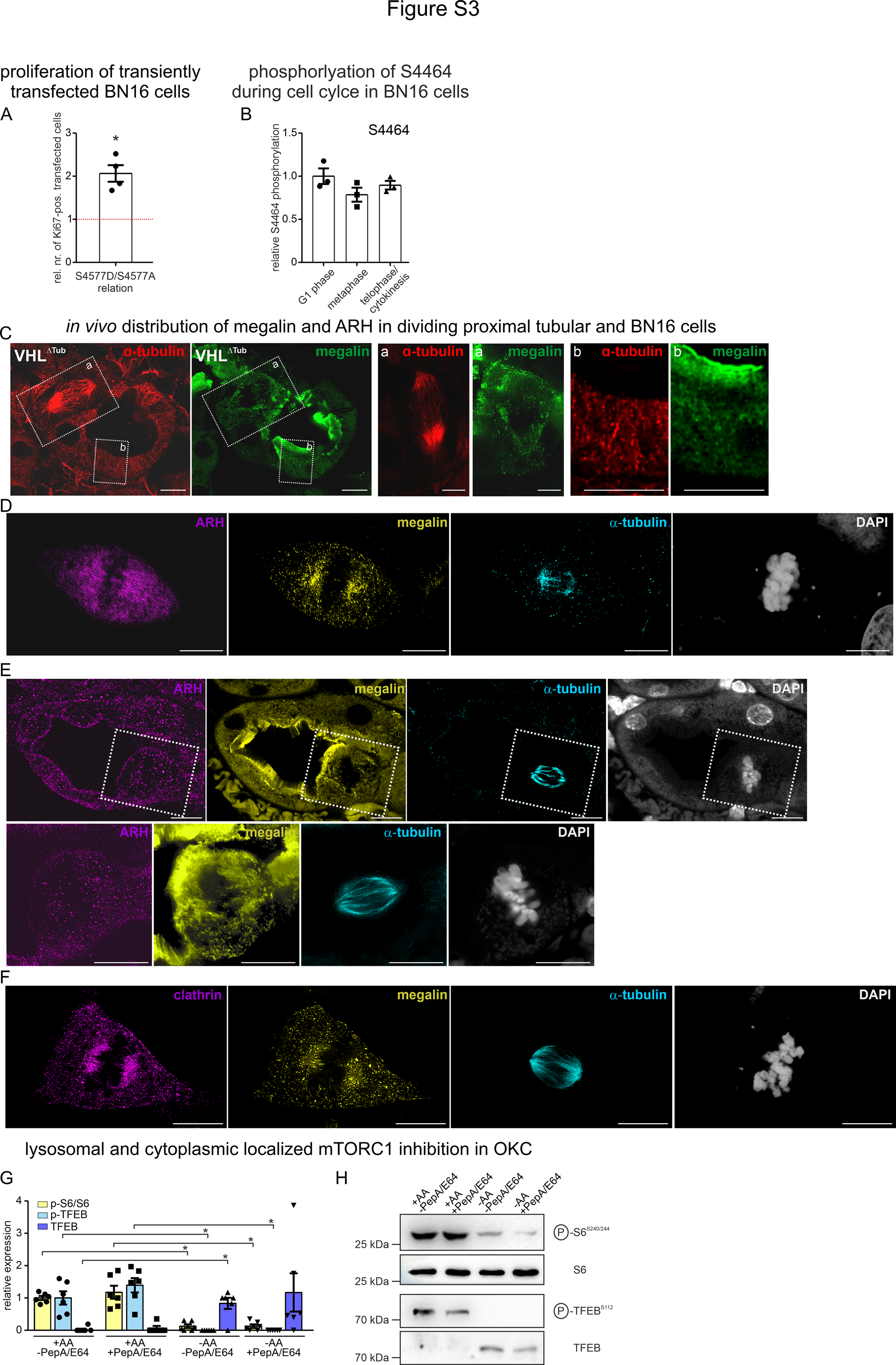

Supplement: Supplementary file 7 — (PNG 2.40 MB) [file 18_2026_6247_Fig11_ESM.png]
