# Supplementary material for: mTORC1-signaling switches megalin function from endocytosis to cell cycle progression
Source: Cell Mol Life Sci. 2026 May 23;83(1):224. doi: 10.1007/s00018-026-06247-5 (PMC13198606; doi:10.1007/s00018-026-06247-5)
Supplement: Supplementary file 9 — Supplementary Material 5 (PDF 4.93 MB) [file 18_2026_6247_MOESM5_ESM.pdf]

supportive original data

Of note: all western blot membranes were cropped before antibody hybridization

belonging to Figure 1C
representative blot

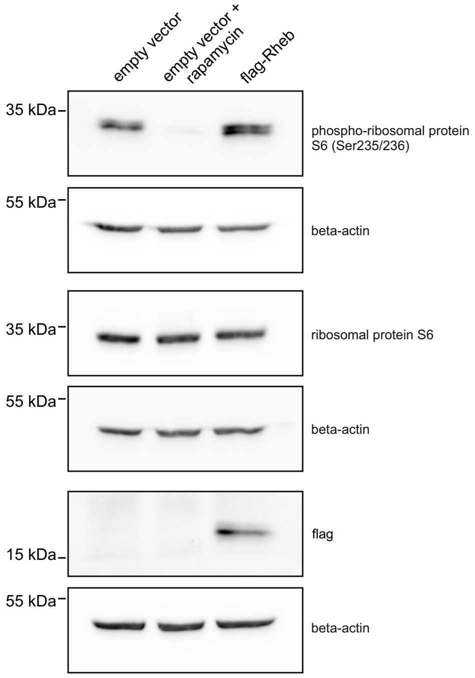

belonging to Figure 1D
quantification

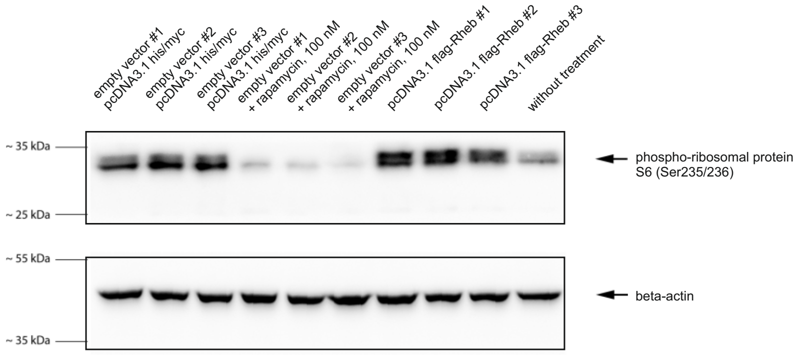

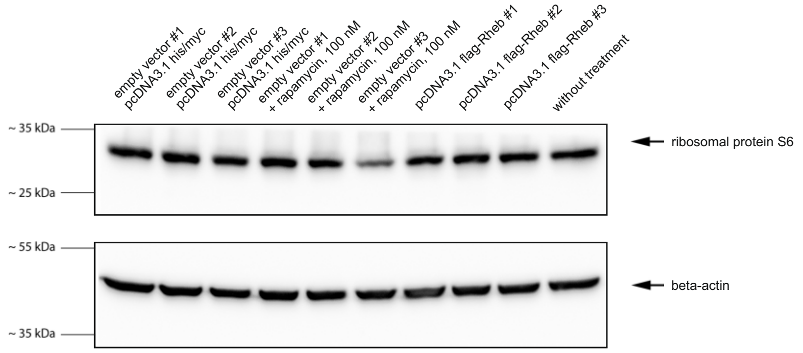

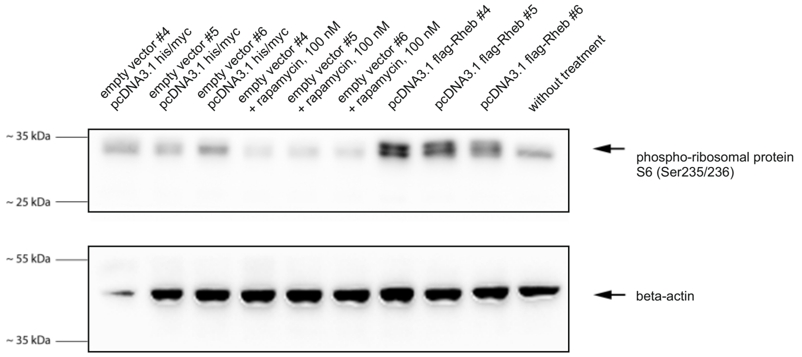

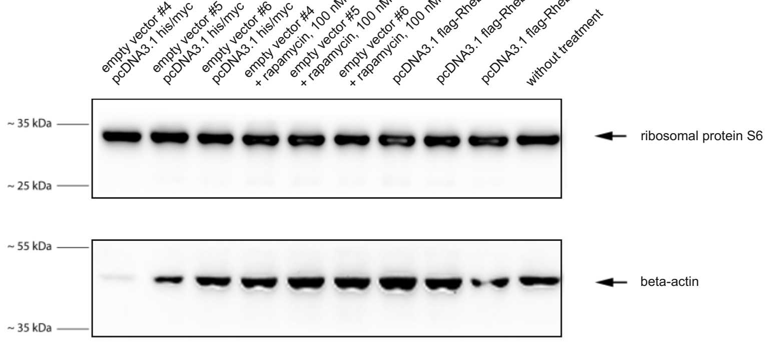

supportive original data

belonging to Figure 2C

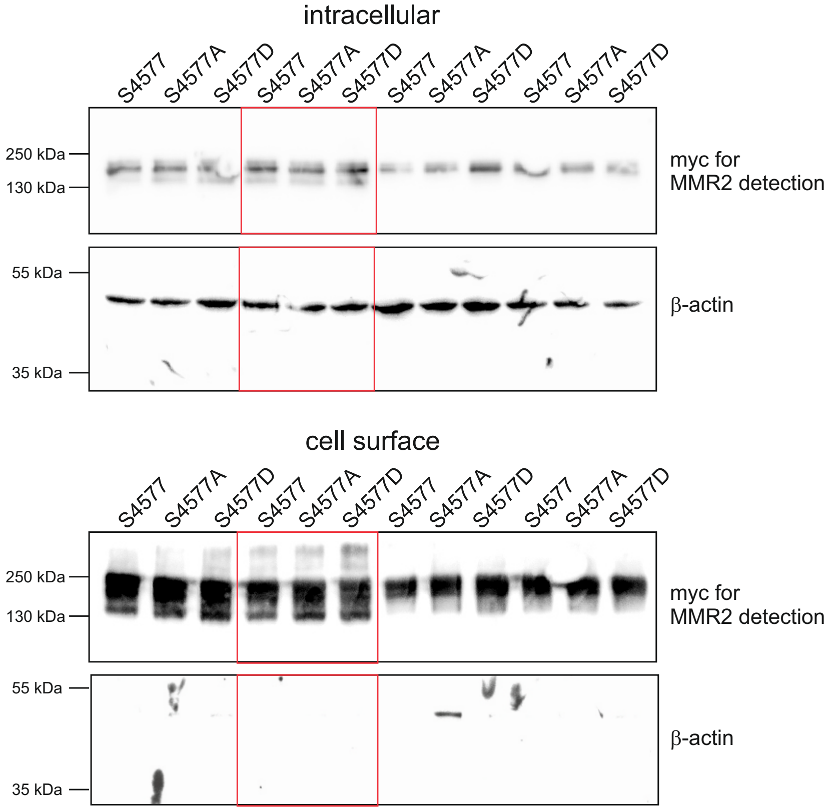

supportive original data

Localisation of myc-tagged MMR2 and megalin in transfected and wt BN16 cells

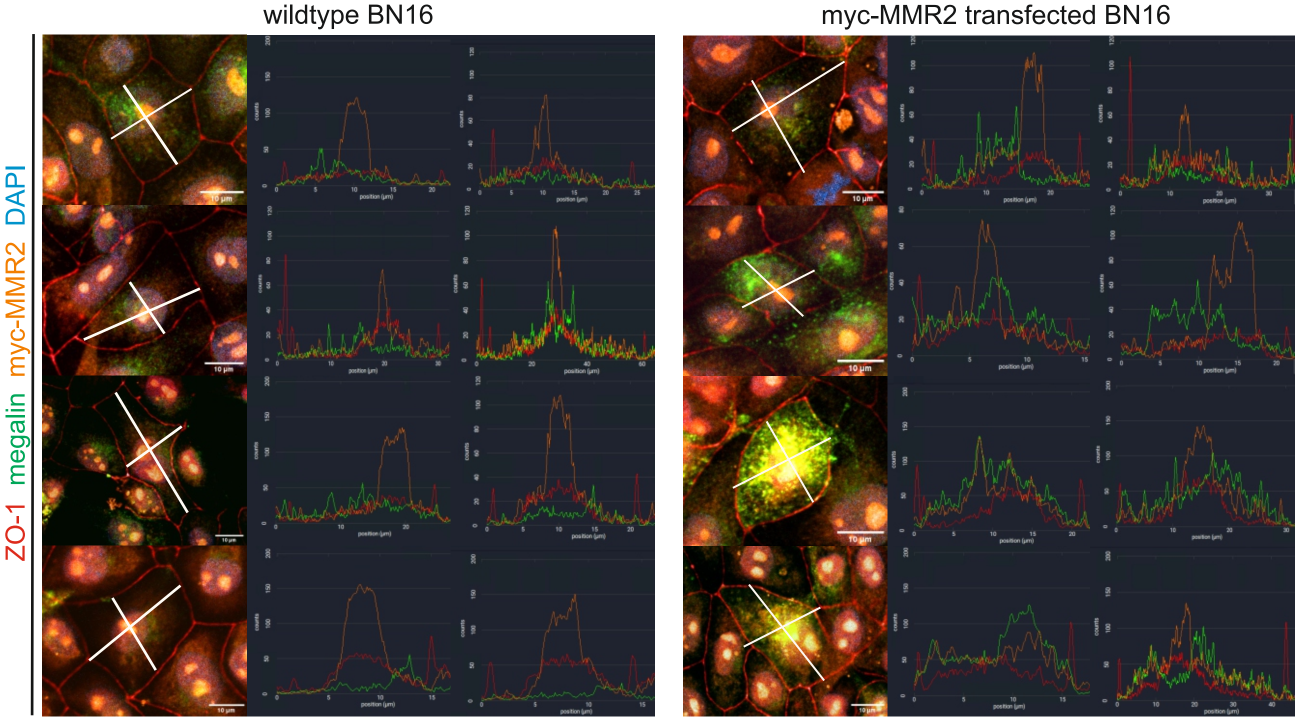

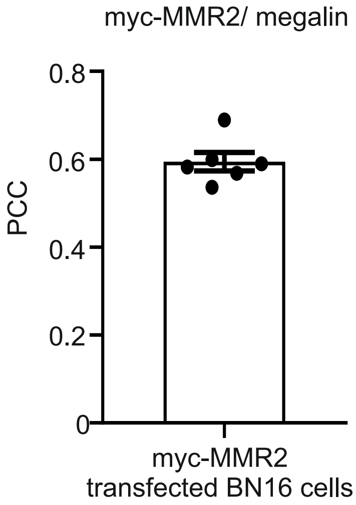

supportive original data

belonging to Figure 4A,B

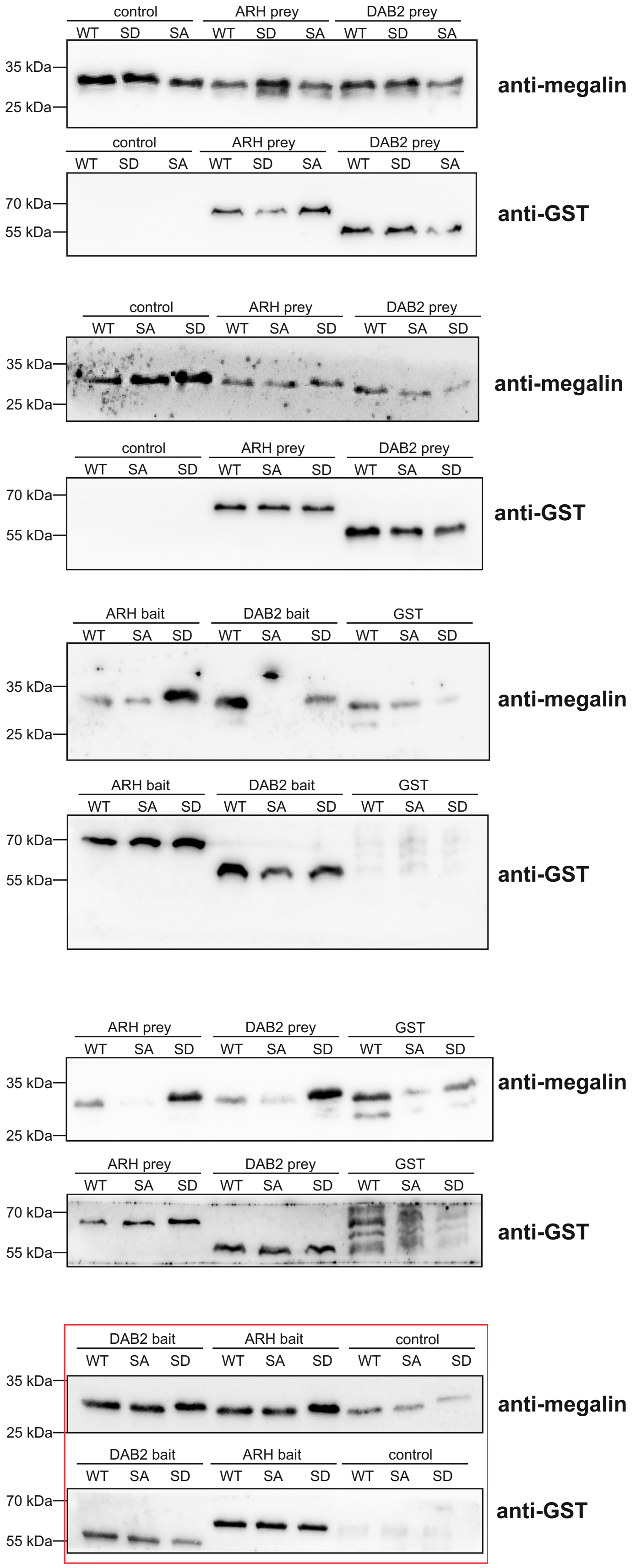

supportive original data

belonging to Figure 4F,G

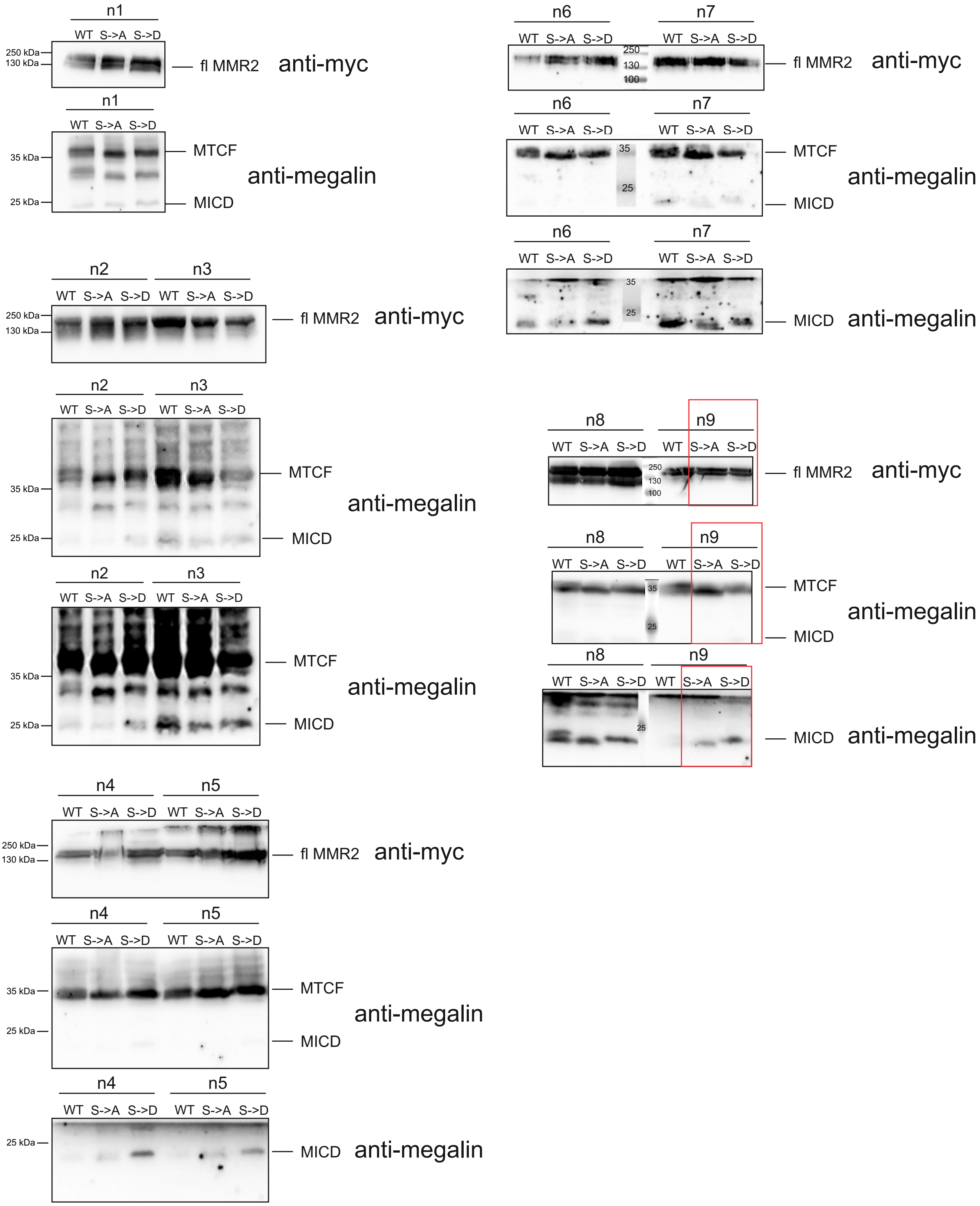

supportive original data

belonging to Figure 5B

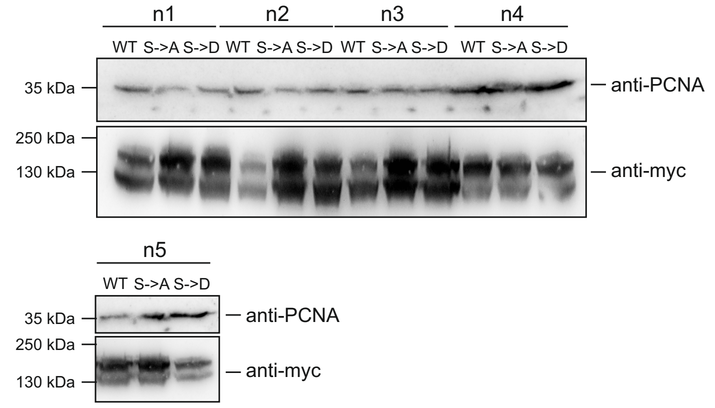

belonging to Figure S3

membranes were cropped before antibody hybridization; loading scheme is repetitive in n1 - n6

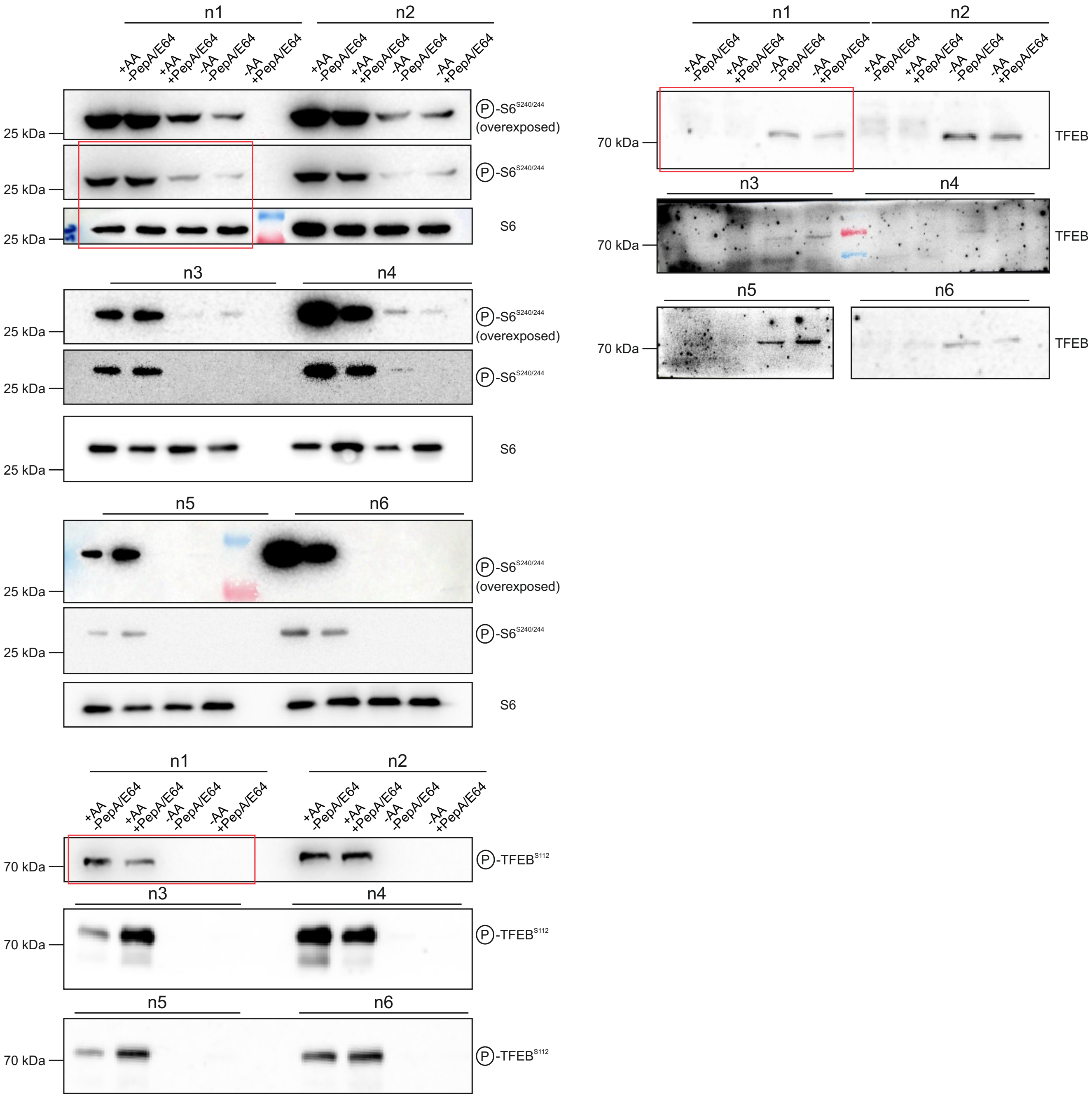

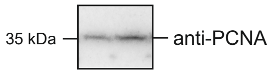
35 kDa
anti-PCNA
